# Linking genome wide RNA sequencing with physio-biochemical and cytological responses to catalogue key genes and metabolic pathways for alkalinity stress tolerance in lentil (*Lens culinaris* Medikus)

**DOI:** 10.1186/s12870-022-03489-w

**Published:** 2022-03-05

**Authors:** Dharmendra Singh, Chandan Kumar Singh, Jyoti Taunk, Kishor Gaikwad, Vijayata Singh, Satish Kumar Sanwal, Sourabh Karwa, Deepti Singh, Parbodh Chander Sharma, Rajendra Kumar Yadav, Madan Pal

**Affiliations:** 1 Division of Genetics, ICAR-Indian Agricultural Research Institute, New Delhi, 110012 India; 2 Division of Plant Physiology, Indian Agricultural Research Institute, New Delhi, 110012 India; 3 ICAR-National Institute of Plant Biotechnology, 110012 New Delhi, India; 4 Division of Crop Improvement, Central Soil Salinity Research Institute, 132001 Karnal, India; 5 Depatment of Botany, Meerut College, 250001 Meerut, India; 6 Department of Genetics and Plant Breeding, Chandra Shekhar Azad University of Agriculture and Technology, 208002 Kanpur, India

**Keywords:** Alkalinity stress, Abscisic acid, Chromosome lesions, Dehydrin, Epigenetics, Lentil, Secondary metabolites

## Abstract

**Background:**

Alkaline soils cause low productivity in crop plants including lentil. Alkalinity adaptation strategies in lentil were revealed when morpho-anatomical and physio-biochemical observations were correlated with transcriptomics analysis in tolerant (PDL-1) and sensitive (L-4076) cultivars at seedling stage.

**Results:**

PDL-1 had lesser salt injury and performed better as compared to L-4076. Latter showed severe wilting symptoms and higher accumulation of Na^+^ and lower K^+^ in roots and shoots. PDL-1 performed better under high alkalinity stress which can be attributed to its higher mitotic index, more accumulation of K^+^ in roots and shoots and less aberrantly dividing cells. Also, antioxidant enzyme activities, osmolytes’ accumulation, relative water content, membrane stability index and abscisic acid were higher in this cultivar. Differentially expressed genes (DEGs) related to these parameters were upregulated in tolerant genotypes compared to the sensitive one. Significantly up-regulated DEGs were found to be involved in abscisic acid (ABA) signalling and secondary metabolites synthesis. ABA responsive genes viz*.* dehydrin 1, 9-cis-epoxycarotenoid dioxygenase, ABA-responsive protein 18 and BEL1-like homeodomain protein 1 had log_2_fold change above 4.0. A total of 12,836 simple sequence repeats and 4,438 single nucleotide polymorphisms were identified which can be utilized in molecular studies.

**Conclusions:**

Phyto-hormones biosynthesis—predominantly through ABA signalling, and secondary metabolism are the most potent pathways for alkalinity stress tolerance in lentil. Cultivar PDL-1 exhibited high tolerance towards alkalinity stress and can be used in breeding programmes for improving lentil production under alkalinity stress conditions.

**Supplementary Information:**

The online version contains supplementary material available at 10.1186/s12870-022-03489-w.

## Background

Alkaline soils affect 434 million hectares of land which cover more than 25% of the earth’s surface (FAO, http://www.fao.org/soils-portal/soil-management). In these soils, plants face a problem of restricted water supply to the roots which is aggravated by the presence of salts like NaHCO_3_ and Na_2_CO_3_ which are more destructive than neutral salts like NaCl (causing salinity) [1]. Also, these soils are highly saturated with CaCO_3_ which limits phosphorous solubility by forming Ca-P compounds, restricting crop productivity in plants [2]. Alkalinity stress triggers acute osmotic stress, ion injury along with high pH (> 8.5) induced injuries [3]. Since third type of injury is not much prevalent in salinity stress, this contributes to higher alkalinity toxicity in plants as compared to the former. High concentrations of HCO_3_ and NaHCO_3_ in alkaline soils effects a wide array of metabolic activities in plants leading to stunted growth, leaf chlorosis and distorted anatomical structures [4]. These massive cyto-morphological disorders occur due to increased Na^+^ uptake, nutritional deficiencies, organic acids imbalance, inorganic ions accumulation, disrupted cellular pH, together with decreased enzyme activities and photosynthetic carbon metabolism [5, 6]. In a comparative study on effect of alkalinity v/s salinity on legume crop *Lathyrus quinquenervius*, it was found that alkalinity stress was much more devastating. It led to higher inhibition of germination, growth, photosynthesis and root system activities. Alkalinity stress also caused organic acid imbalance, increased H_2_O_2_ and malondialdehyde (MDA) contents, ultimately leading to morphological impairment in this leguminous forage [7].

In order to mitigate alkalinity stress, it is necessary to dissect key genes and metabolic pathways for development of stress tolerant germplasm which can be utilized for breeding purposes. Previous studies indicated that complex molecular mechanisms are associated with tolerance towards NaHCO_3_ or Na_2_CO_3_ toxicities in crop plants [8]. Manipulation of molecular components including iron transporters, iron reductases and other enzymes have resulted in production of transgenic plants with improved tolerance against low-Fe availability with higher grain yield in calcareous soils [9, 10]. Some alkalinity tolerant and/or sensitive mutants, such as *pks5* and *j3* in *Arabidopsis* [11]; and *alt1* in rice [12] have been isolated and characterized. The mutant chaperone J3 positively regulated alkalinity tolerance by interacting with protein kinase, PKS5 to increase plasma membrane H^+^-ATPase activity in *Arabidopsis* [11]. In rice, mutant ALT1, a putative Snf2 family chromatin remodelling ATPase showed enhanced alkalinity tolerance through substantial defence against oxidative stress [12].

Lentil (*Lens culinaris* Medikus) is a leguminous plant, cultivated throughout Europe, Asia and North America for its highly proteinaceous seeds which serve as staple food for humans and its straw as animal fodder. Furthermore, its ability of nitrogen fixation and carbon sequestration improves soils’ nutrient status and thus helps in agricultural sustainability [13]. Globally, it is cultivated on 4.8 Mha with a production of 5.7 MT (FAO STAT, 2019). Lentil is moderately sensitive to both alkalinity and salinity stresses. However, two cultivars namely, PDL-1 and PSL-9 were found to be tolerant against both the stresses [4, 14]. These cultivars have already been released in India to be cultivated under salt affected soils. Reduced Na^+^ content and increased K^+^ content was found to be associated with tolerance of these cultivars under salinity and alkalinity stress conditions [4, 14].

At the cellular level, tolerant plants can compartmentalize Na^+^ in vacuoles resulting in increase in their tolerance towards high concentration of ions. Evidently, PDL-1 showed lower concentration of Na^+^ and higher concentration of K^+^ in both roots and shoots under long term exposure (15 d) of alkalinity stress (40 mM NaHCO_3,_) as compared to the sensitive cultivar, L-4076 [4]. PDL-1 has also been registered as a drought tolerant cultivar at National Bureau of Plant Genetic Resources, New Delhi, India in 2017. This cultivar has also showed tolerance against heat stress [15]. Thereby, the cultivar poses to be the most promising lentil cultivar for identifying genes associated with various abiotic stress tolerances including alkalinity.

In last one decade, some attempts have been made to identify genes responsible for alkalinity stress tolerance in crops like sorghum, soybean, jujube, rice, wheat, sugar beet, etc. [16–21]. Most of these studies utilized Ribonucleic acid (RNA)-sequencing (RNA-seq) approach to identify alkalinity responsive genes. RNA-seq is a highly powerful tool for accurate characterization of gene-expression, even at a single nucleotide level under varied stress conditions. However, investigation of stress responsive genes using RNA-seq is limited in lentil. Few studies have utilized this approach to identify drought [22], heat [23] and aluminium (unpublished data) stress responsive genes in lentil. However, exhaustive evaluation of alkalinity responsive genes and its gene regulatory pathways is entirely missing in case of lentil. Therefore, the present investigation was undertaken to i) dissect morpho-anatomical and physio-biochemical changes under alkalinity stress; ii) deduce differentially expressed genes (DEGs) associated with different metabolic pathways involved in response to alkalinity stress; iii) discover simple sequence repeats (SSRs) and single nucleotide polymorphisms (SNPs) among the contrasting cultivars. This investigation will provide deeper insight for genetic improvement of alkalinity stress tolerance in lentil.

## Results

### Morpho-anatomical responses under alkalinity stress

**Fig. 1:**
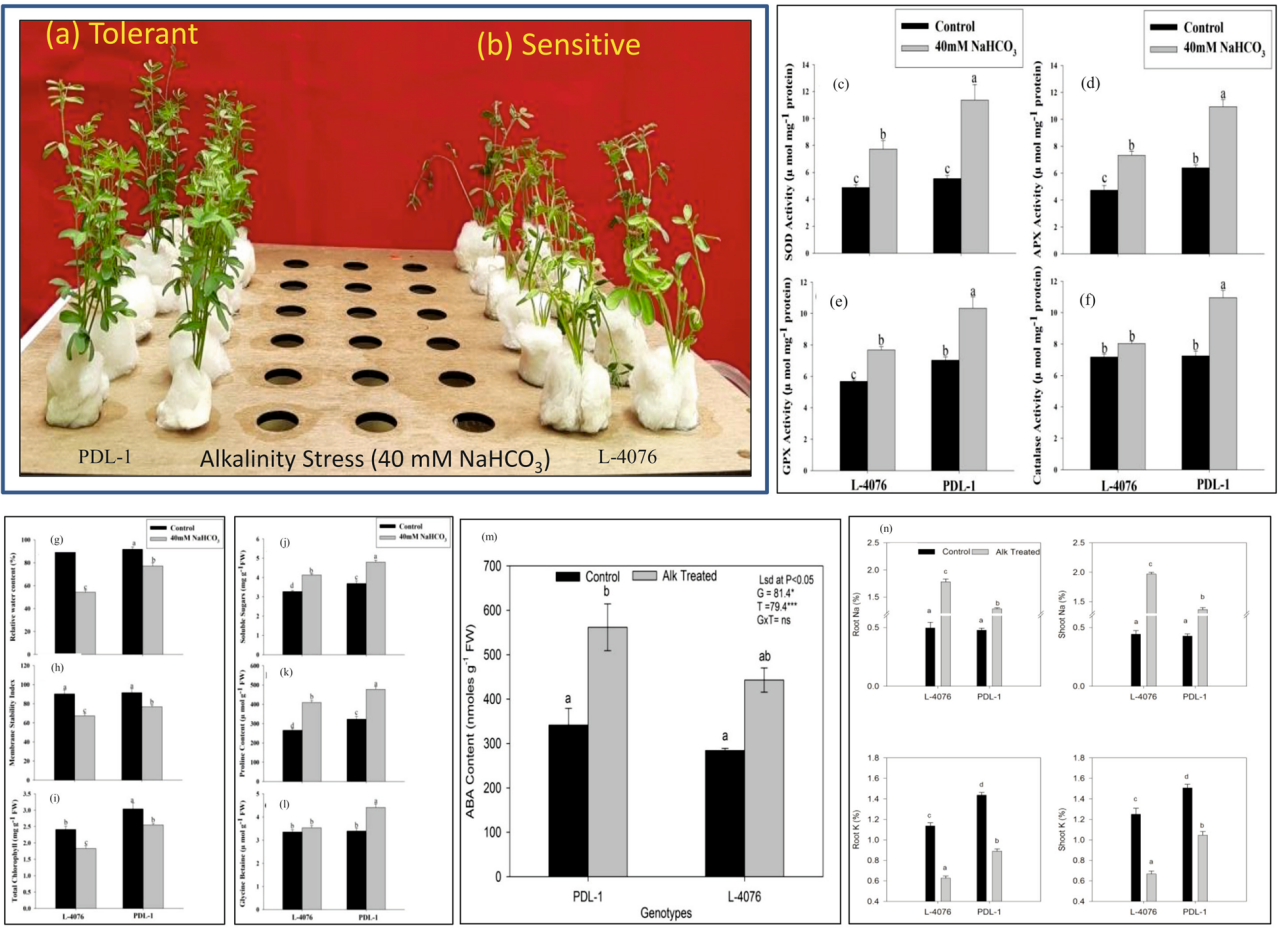
Morphological response of alkalinity **a**) tolerant (PDL-1) and **b**) sensitive (L-4602) cultivars of lentil under 40 mM NaHCO_3_ stress in hydroponics. Bar graph represents antioxidant enzyme activities of **c**) Superoxide Dismutase (SOD) **d**) Ascorbate Peroxidase (APX) **e**) Glutathione Peroxidase (GPX) **f**) Catalase; physiological responses in terms of **g**) Relative Water Content (RWC) **h**) Membrane Stability Index (MSI), **i**) Total Chlorophyll content; osmolyte accumulation of **j**) soluble sugar, **k**) proline, **l**) Glycine betaine; phyto-hormone activity of **m**) Absicisic acid (ABA) along with **n**) root-shoot sodium and potassium contents for both the cultivars under control and alkalinity stress conditions

Two contrasting lentil cultivars viz. PDL-1 (tolerant) and L-4076 (sensitive) exhibited different response against alkalinity stress. Alkalinity stress symptoms increased in leaves with increase in time duration (3, 5 and 7 d) in 40 mM NaHCO_3_. Seedlings of sensitive cultivar exhibited severe wilting whereas tolerant cultivar did not show such symptoms even on the fifth day of the alkalinity stress (Fig. 1a, b). This clearly indicates that the most prominent visible differences between tolerant and sensitive cultivars were evident on the fifth day of alkalinity exposure (Fig. 1 a, b). Therefore, 5 days time length was considered enough to check initial differences against alkalinity stress and thus the same time period was selected for further physio-biochemical, anatomical and transcriptomic analyses in this study. Data obtained from these analyses revealed that PDL-1 has high tolerance against alkalinity stress and the results are in line with our previous observations [4].

**Fig. 2:**
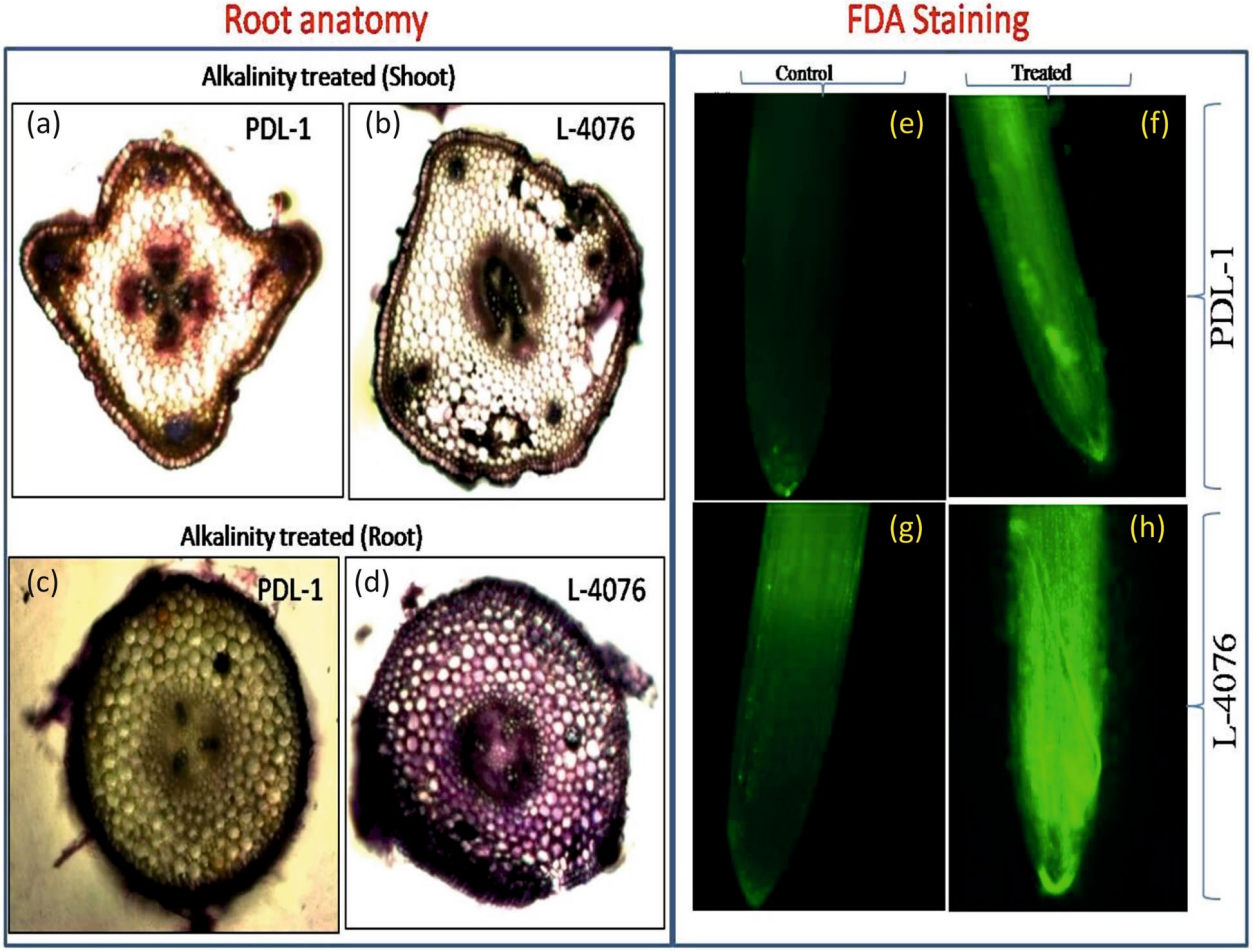
Free hand cross sections of lentil **a**, **b**) shoot **c**, **d**) root stained with toulidine blue and **e**–**h**) H_2_O_2_ detection in roots using Fluorescein diacetate (FDA) for tolerant (PDL-1) and sensitive (L-4076) cultivars under control and alkalinity stress conditions

Under control condition, tolerant and sensitive cultivars displayed a well-organized structure of their roots and shoots characterized by intact epidermal cells, multiple layers of cortex tissue and parenchymatous cells (Fig. 2 a-d). Root and shoot anatomy under 40 mM NaHCO_3_ treated condition showed that the tolerant cultivar maintained good organization of inner root and shoot structures, whereas sensitive cultivar showed disintegration of internal structure along with distortion of root and shoot architectures (Fig. 2 a-d). Thus, the cellular integrity in the tolerant cultivar was much higher as compared to the sensitive one (Fig. 2 a-d). Further, Fluorescein diacetate (FDA) fluorescence signal was increased in roots of both sensitive and tolerant cultivars under stress condition when compared to their respective controls (Fig. 2 e–h). However, the intensity of fluorescence signal was much lower in PDL-1 than L-4076 under alkalinity stress (Fig. 2 f, h).

### Physio-biochemical responses under alkalinity stress

Variability in physiological parameters; antioxidant enzyme activities viz. superoxide dismutase (SOD), ascorbate peroxidase (APX), glutathione peroxidase (GPX) and catalase (Fig. 1 c-f) and accumulation of compatible solutes increased in both the cultivars under alkalinity stress (Fig. 1 g-l). However, antioxidant enzyme activities and accumulation of proline, glycine betaine, soluble sugars, relative water content (RWC), and membrane stability index (MSI) were higher in the tolerant cultivar (PDL-1) as compared to the sensitive one (L-4076) (Fig. 1 g-l). Significant increase in abscisic acid (ABA) content was noticed in both the cultivars under alkalinity stress condition when compared with their controls. However, the content was slightly higher in tolerant cultivar (PDL-1) (Fig. 1 m).

Na^+^ and K^+^ contents in roots and shoots of lentil plants were also measured to find out their physiological response. Na^+^ content in roots and shoots of tolerant (PDL-1) and sensitive (L-4076) cultivars was significantly increased under alkalinity stress (Fig. 1 n). After the 5^th^ day of stress, L-4076 had higher Na^+^ content in both roots and shoots than PDL-1. In contrast, K^+^ contents were dramatically reduced after stress treatment; while PDL-1 had higher K^+^ content in roots and shoots as compared to L-4076. This shows that higher alkalinity tolerance of PDL-1 might be associated with its low accumulation of Na^+^ and high accumulation of K^+^ in both roots and shoots (Fig. 1n).

### Cytological changes under alkalinity stress

**Fig. 3:**
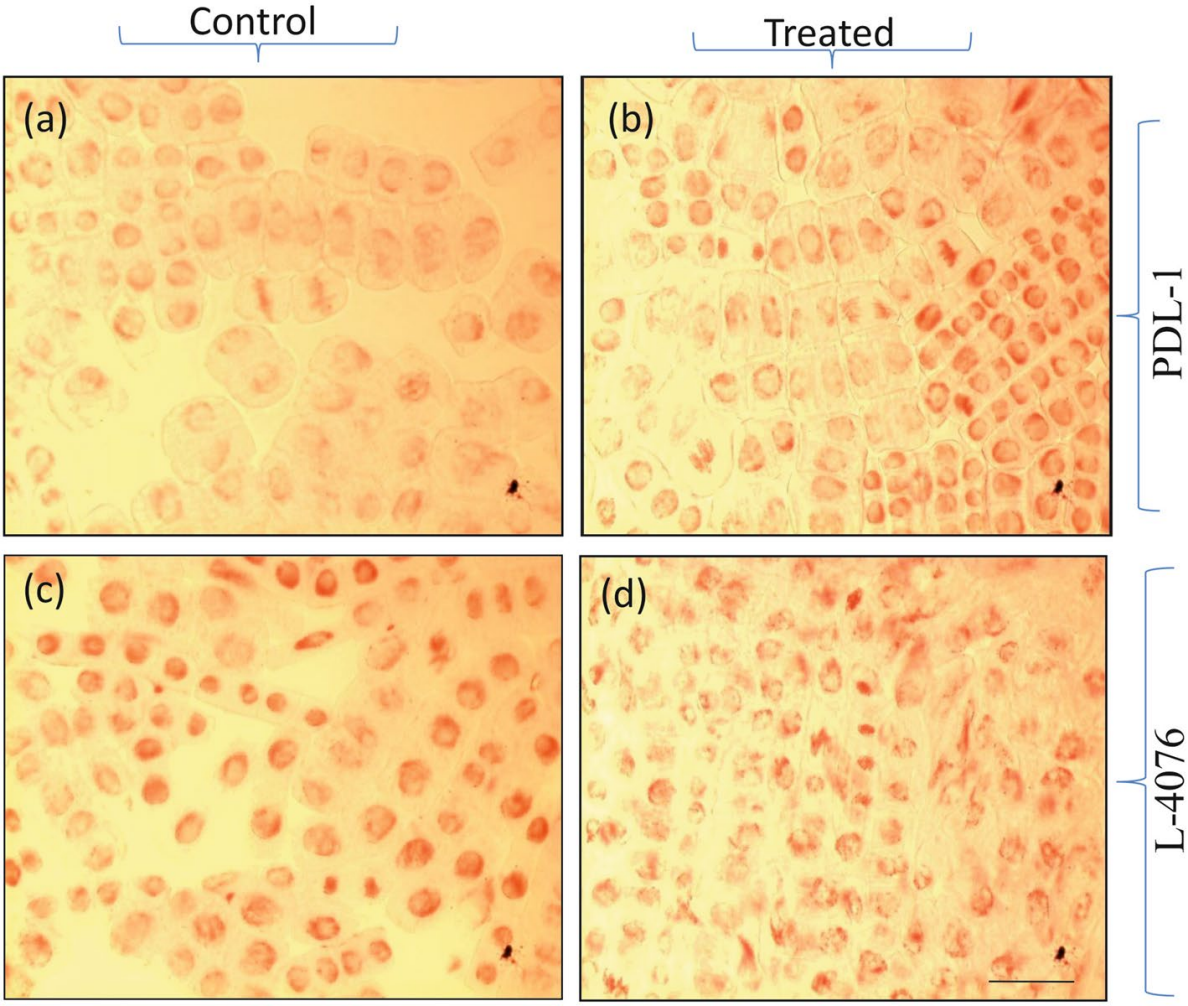
Microscopic field view of dividing lentil root tip cells of **a**-**b**) tolerant and **c**-**d**) sensitive cultivars under control and alkalinity stress conditions after 24 h

Mitotic index (MI) was found higher in both the cultivars under control condition as compared to the alkalinity stress condition after 24 h (Table S1, Fig. 3). Under control condition, MI of sensitive cultivar (L-4076) was much higher than tolerant cultivar (PDL-1). However, with increasing time, chromosomal aberrations and atypically dividing cells were more evident in this cultivar. On the other hand, MI of tolerant cultivar was more under stress condition than that of sensitive cultivar. Moreover, under stress condition, average number of atypically dividing cells was more in case of L-4076 as compared to PDL-1 (Fig. 3 b, d).

### De novo assembly and functional annotation of reads

Average paired-end reads generated through whole transcriptome sequencing for the sample 1C, 2C, 1 T and 2 T, including their biological replicates were 57,893,435, 62,542,645, 30,757,579 and 19,605,802 bp, respectively, where 90.65 to 92.41% of total reads were mapped. Average number of contigs generated under control and treated samples in contrasting cultivars i.e. PDL-1 and L-4076 were 40,386 (1C), 42,936 (2C), 28,596 (1 T) and 19,730 (2 T) bp while the longest contig lengths were 19,703 (1C), 15,591 (2C), 15,527 (1 T) and 15,305 (2 T) bp. Average N_50_ for these samples were 1749 (1C), 1774 (2C), 1176 (1 T) and 1055 (2 T) bp (Table S2).

### Differential response of genes under alkalinity stress

**Fig. 4:**
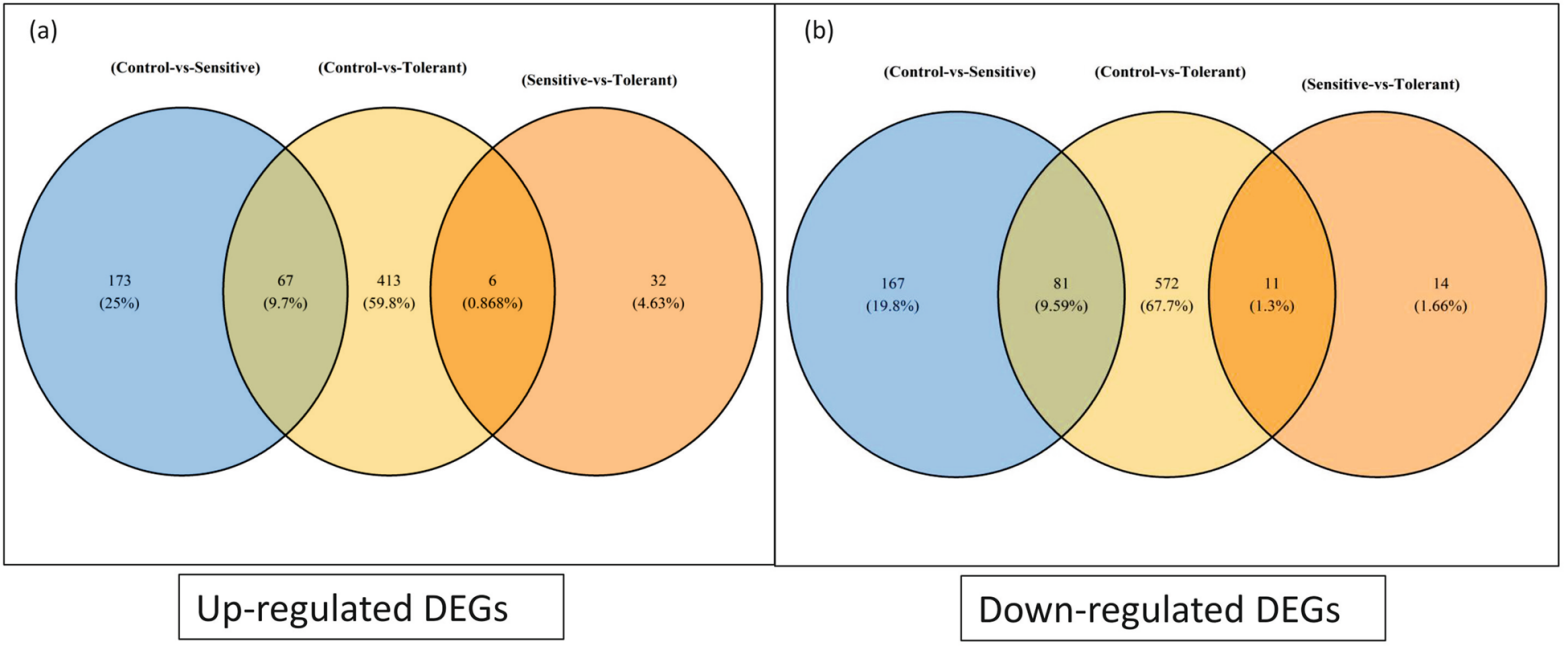
Venn diagram representing **a**) up-regulated and **b**) down-regulated differentially expressed genes (DEGs) in different combinations viz. 1C-1 T, 2C-2 T and 1 T-2 T where 1C: PDL-1 control, 1 T: PDL-1 treated, 2C: L-4076 control and 2 T: L-4602 treated

Venn diagram showing total number of up and down regulated DEGs under different combinations is represented in Fig. 4. These DEGs were further sorted following the criteria of adjusted *p-*value ≤ 0.05 and log_2_fold change > 1.5. A total of 1150 genes were assorted which comprises 486 up-regulated and 664 down-regulated DEGs in the combination 1 T vs 1C. Further, 38 up-regulated and 25 down-regulated DEGs were observed in the combination 1 T vs 2 T. In sensitive cultivar L-4076, a total of 240 up-regulated and 248 down-regulated unigenes were found between control and treated conditions.

### Differentially expressed alkalinity responsive genes

Several alkalinity responsive genes which were differentially expressed under control and treated conditions in contrasting cultivars were identified. Most of these genes were found to be involved in cell cycle regulation, signal transduction, export pathways, together with proteins, carbohydrates and lipids formation. Putative alkalinity-stress responsive genes with highest log_2_fold changes are listed in Table S3.

### Differentially expressed alkalinity tolerance genes

**Fig. 5:**
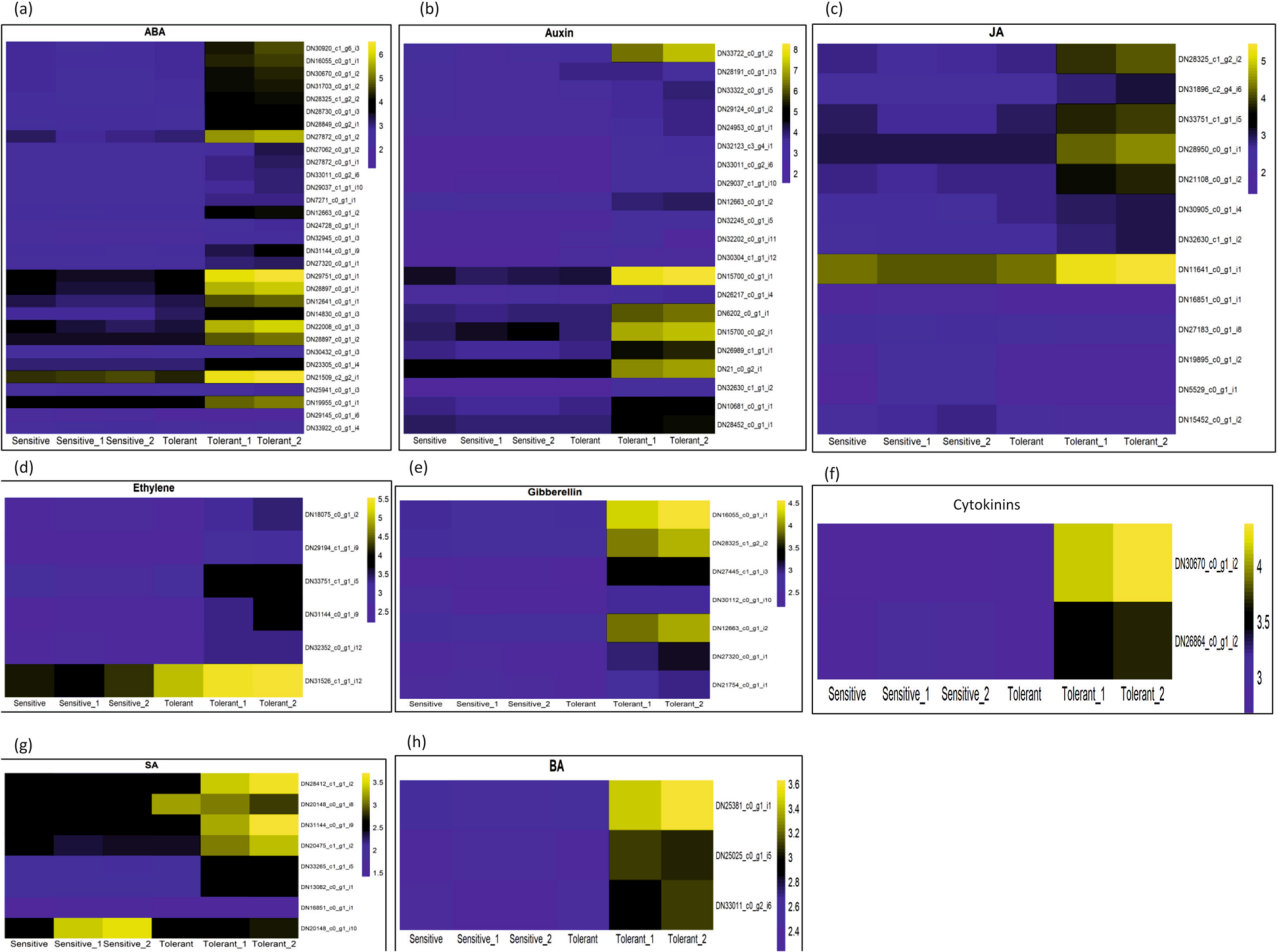
Heat maps showing differential expression of phyto-hormone related differentially expressed genes (DEGs) under alkalinity stress for **a**) absicisic acid (ABA), **b**) auxin, **c**) jasmonic acid (JA), **d**) ethylene, **e**) gibberellin, **f**) cytokinins, **g**) salicylic acid and **h**) Brassinosteroids (BR) in alkalinity tolerant and sensitive lentil cultivars. DN33722_c0_g1_i2 in part **b**) represents Auxin transport protein BIG which has been validated in qRT-PCR. First three columns of heat map represent replicates of sensitive cultivar (L-4076), whereas last three columns represent replicates of tolerant cultivar (PDL-1)

In order to identify possible alkalinity tolerance candidate genes in lentil, DEGs between alkalinity treated contrasting cultivars (PDL-1 v/s L-4076) were filtered out. Twenty-two DEGs were found to be associated with root-shoot growth and development. Seven DEGs were involved in root nodule formation, whereas 5 DEGs were related to chlorophyll biosynthesis. Thirty-two DEGs were involved in inter-membrane trafficking and microtubules movement. Seventeen DEGs were found to be associated with antioxidant compounds and secondary metabolism. Twenty seven DEGs were responsible for reconciliation of mitosis and cell division, whereas 21 DEGs adhere to orchestration of epigenetic responses. A total of 12, 118 and 134 DEGs were related to cell wall; ion channels and transporters; and intracellular signalling mediated by calcium, lipids, transcription factors, and phyto-hormones; respectively. Heat maps showing DEGs related to phyto-hormones that were found to be up-regulated in tolerant cultivar (1 T) under alkalinity stress are represented in Fig. 5. Categorical representation of top up and down regulated DEGs along with their associated functions in alkalinity stress response is summarized in Table S4. Circos plots were also generated for up and down-regulated DEGs between contrasting cultivars under control and treated conditions (Fig. S1).

## SNP and SSR calling

A total of 12,836 SSRs were identified in this investigation, of which 2C had the maximum number of SSRs i.e., 7860 followed by 1C which had 7299 SSRs. Under alkalinity stress conditions, 1 T showed more SSRs (3300) compared to 2 T which had only 2032 SSRs. Total number of SNP variants identified within samples of tolerant and sensitive cultivars under treated conditions were 933 and 1146, respectively.

## Pathway analysis

**Fig. 6:**
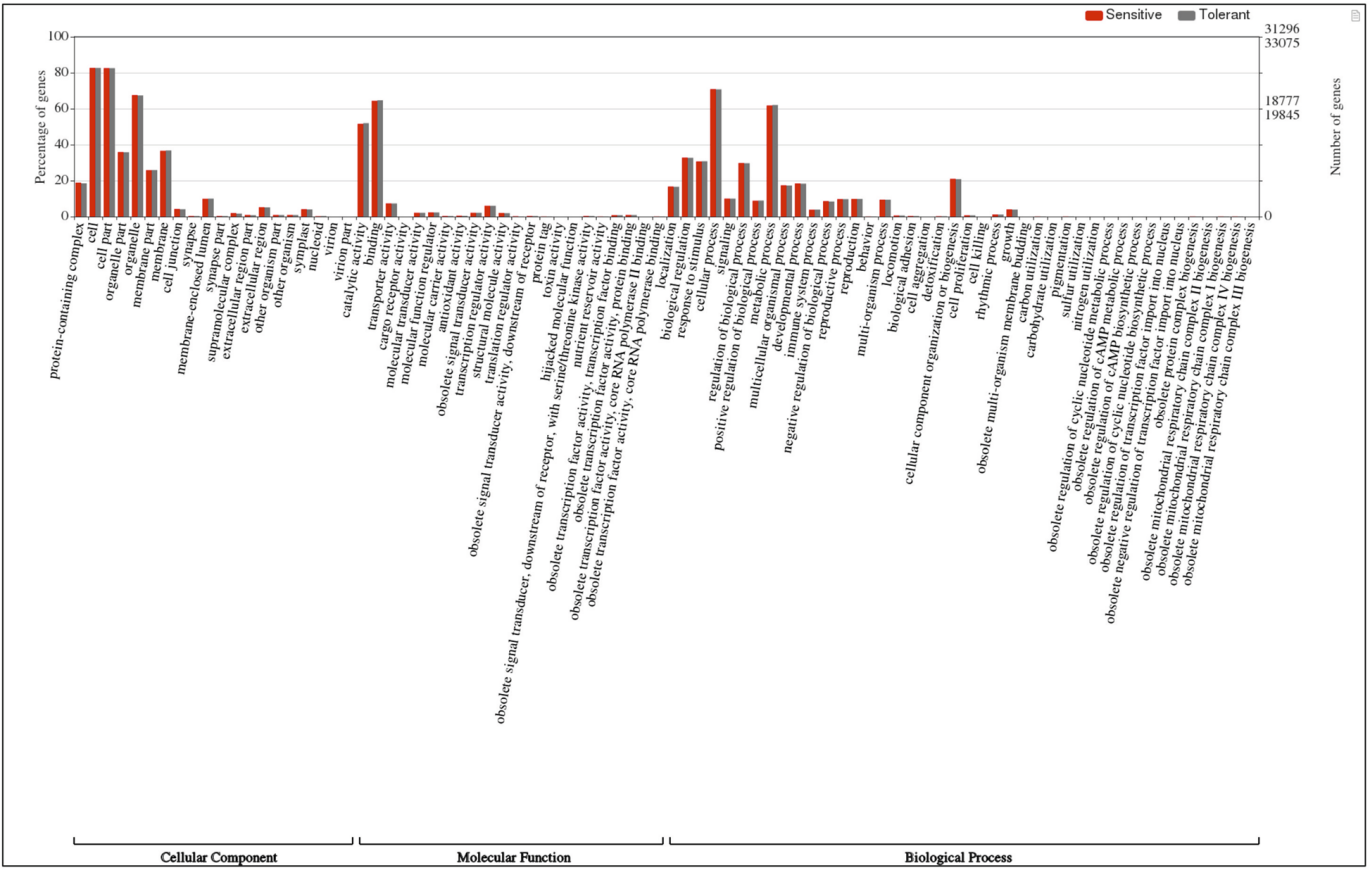
Web Gene Ontology Annotation (WEGO) plot representing gene percentage sharing of differentially expressed genes (DEGs) in tolerant and sensitive cultivars of lentil under alkalinity stress that were categorised for cellular component, molecular function and biological processes

**Fig. 7:**
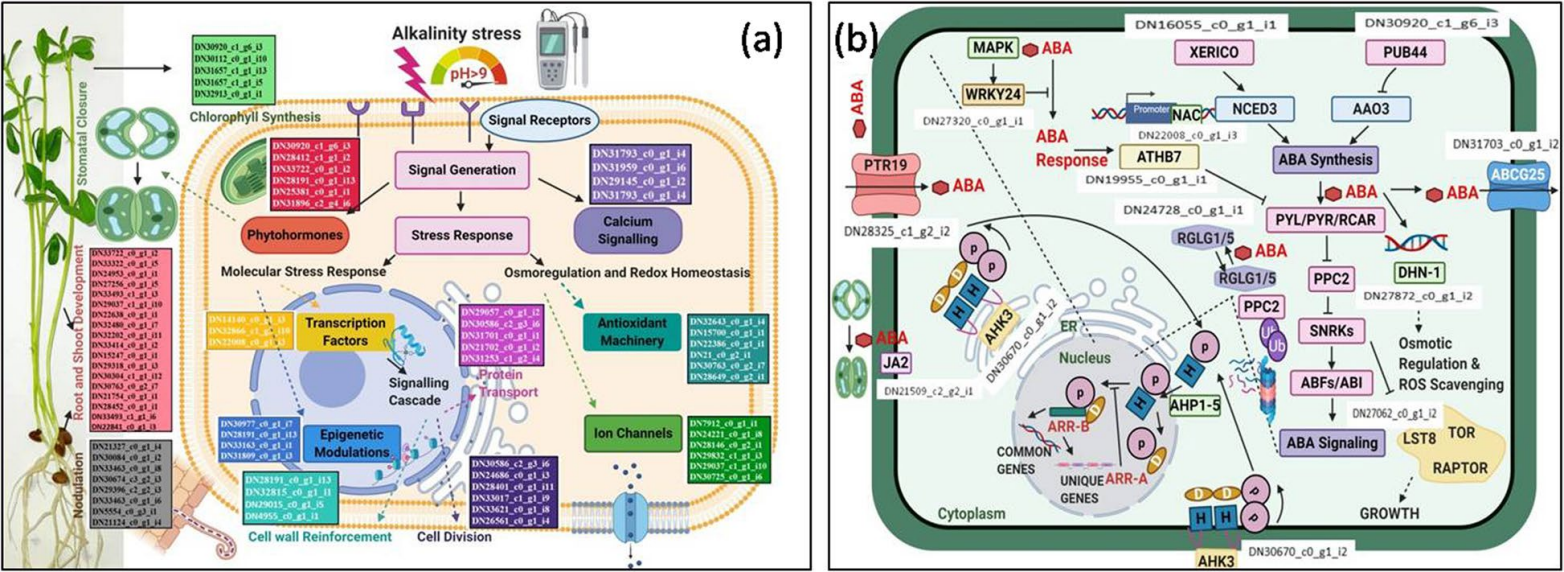
Schematic representation of (**a**) overall putative differentially expressed genes (DEGs) and modulated pathways in response to alkalinity stress, **b**) Detailed description of DEGs related to ABA pathways which were induced due to alkalinity stress. PUB44: Promotes the degradation of AAO3 and thus represses abscisic acid (ABA) biosynthesis; XERICO: Function on ABA homeostasis at post-translational level, probably through ubiquitin/proteasome-dependent substrate-specific degradation; AHK3: Redundant negative regulator of ABA signalling; ABC25G: High affinity ABA transporter that mediates export of ABA, with a preference for ( +)-ABA, through the plasma membrane, especially in vascular tissues (e.g. phloem companion cells), and is involved in the intercellular ABA signalling pathway; PTR19: Mediates cellular ABA uptake; DHN1: Induced by ABA; LST8: Involved in plant growth; RGLG5: Mediates ubiquitination and subsequent proteasomal degradation of the target protein PP2CA, a major inhibitor of ABA signalling; WRKY24: Negative regulator of ABA signalling via specific repression of ABA-induced promoters; NAC2: Transcription factor that activates the expression of senescence and ABA associated genes including NCED1, ABCG40, CYP707A2, SAG113, SGR1 and PAO, by directly binding to their promoters; JA2: Regulates the expression of NCED1, and required for the stomatal closure. Both the images are created with BioRender.com

All the unigenes detected in this study were categorized into Gene Ontology (GO) terms, based on nucleotide sequence similarity (Fig. 6). These GO terms were further broken down into three major categories consisting of biological processes, cellular components and molecular functions. Out of the three major categories, highest number of unigenes belonged to the biological processes (Fig. 6). MapMan based analysis revealed alterations in metabolic pathways that occur during alkalinity stress (Fig S2). Metabolic overview map of sensitive v/s tolerant treated cultivars elucidated that antioxidant machinery, glycolysis, large enzyme families, lipid biosynthesis, mitochondrial electron transport, sucrose synthesis, phyto-hormones and secondary metabolism were the major pathways affected by alkalinity stress. Genes involved in phyto-hormones and secondary metabolites biosynthesis pathways i.e. mevalonic acid (MVA) pathway, phenolics, lignins, and flavonoid synthesis pathways, etc. were found to be affected by alkalinity stress in both the cultivars (Fig S2). Results observed through MapMan based analysis were in compliance with the transcriptomic analysis. Transcriptome data reported multiple DEGs related to carbohydrate anabolism and catabolism reflecting altered glycolysis in MapMan analysis. Similarly, multiple DEGs related to redox homeostasis, cell wall modifications, phyto-hormone synthesis, signalling and secondary metabolites production were also represented in MapMan analysis. Schematic representation of putative DEGs and pathways involved in response to alkalinity stress is represented in Fig. 7 (a). Present study also emphasized upon the induction of ABA related DEGs under alkalinity stress. Thereby, detailed schematic representation of all the DEGs related to ABA pathways is presented in Fig. 7 (b).

## Quantitative real time polymerase chain reaction (qRT-PCR) validation

**Fig. 8:**
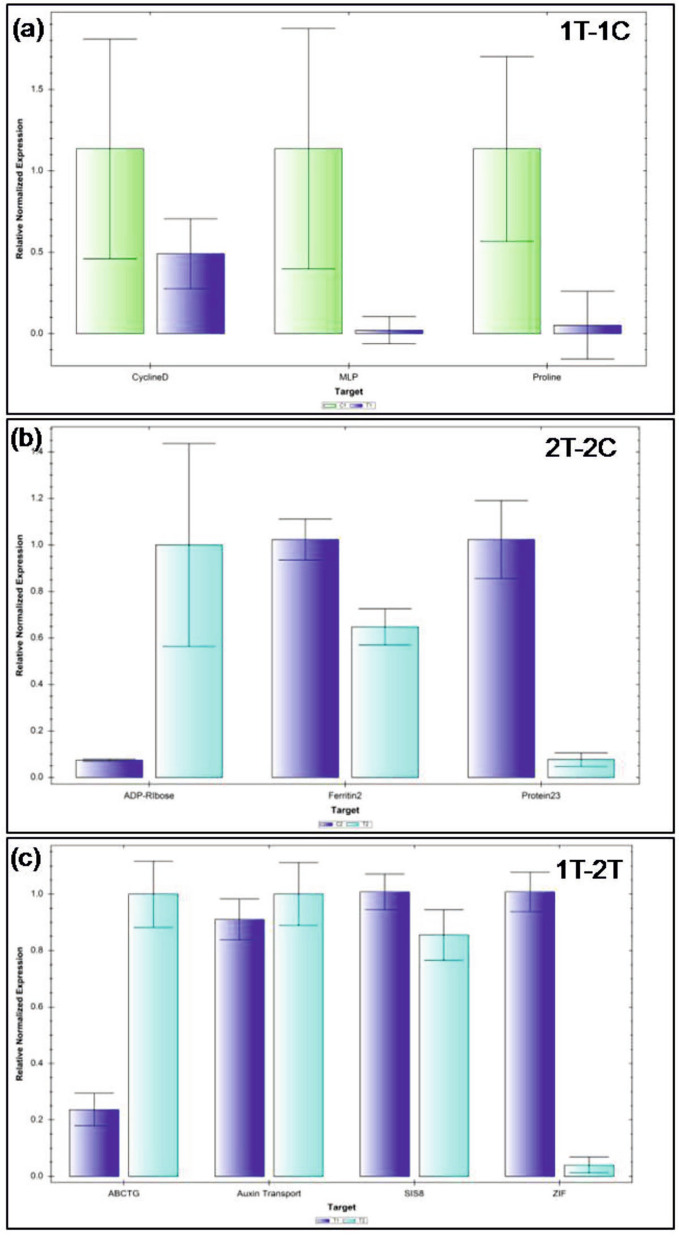
Bar graph representing relative expression of 10 selected differentially expressed genes (DEGs) using quantitative real-time -polymerase chain reaction (qRT-PCR) in different combinations i.e. 1C-1T, 2C-2T, 1T-2T to validate next-generation sequencing (NGS) results of lentil cultivars under alkalinity stress conditions, where 1C: PDL-1 control, 1T: PDL-1 treated, 2C: L-4076 control and 2T: L-4602 treated

In order to validate the results obtained from transcriptomic analysis, qRT-PCR analysis was undertaken using 10 significant genes (Table S5). The relative expression of these DEGs under different combinations is represented in Fig. 8. The observed fold change in qRT-PCR expression results were in accordance with the transcriptomic data, although differences were noted in the absolute expression levels as represented in Fig. 8. Regression plot showed that expression data of next generation sequencing (NGS) and qRT-PCR were found to be highly correlated (Fig S3).

## Discussion

Alkalinity stress is one of the environmental factors that cause physiological stress in plants limiting plant growth and productivity together with generating negative effects on grain quality parameters [24, 25]. Previously, we reported that alkalinity stress considerably reduced plant growth in 285 lentil genotypes at the seedling stage. Also, the seed yield of these genotypes was reduced by 27–87% and 83–100% under alkaline field conditions having soil pH of 9.0 and 9.5, respectively [4]. Based on their morpho-physiological responses, the study also clustered these genotypes into contrasting groups for alkalinity stress tolerance. Following this analysis, two highly contrasting genotypes i.e. PDL-1 and L-4076 were selected for the present study to decipher putative genes and pathways involved in response to alkalinity stress. The selected cultivars have already been used as contrasting cultivars in molecular mapping of salt [14], heat [15] and drought [26] tolerance gene(s), since in our preliminary molecular diversity studies, they were clustered into contrasting groups when analysed under respective stress conditions [14, 15, 22].

In the present study, different physio-biochemical, cytological and molecular analyses were conducted during seedling stage, since in most of the leguminous crops, this stage is considered to be very sensitive as compared to other developmental stages under different abiotic stresses and the stage is critical for plant growth and overall survival [27–29].

### DEGs associated with morpho-anatomical and physio-biochemical parameters under alkalinity stress

Wilting and necrosis are common symptoms of morphological damage due to excess of salt [30]. By the end of fifth day of the treatment, severe wilting and necrosis were predominant in L-4076 seedlings under alkalinity stress which was not the case with tolerant cultivar, PDL-1. Similar results were also obtained in our previous study under long term alkalinity stress [4]. Plants maintain their alkalinity tolerance mechanism by preventing K^+^ efflux and promoting Na^+^ efflux from the cells to maintain their membrane potential [31, 32]. Similarly, in the present study, high alkalinity tolerance of PDL-1 can be linked to its capability to accumulate higher K^+^ content in both roots and shoots as compared to L-4076 (Fig. 1 n).

FDA staining of root tips can detect H_2_O_2_ levels under alkalinity stress, which directly corresponds to the intensity of fluorescent signals. In a study by Biswas and Mano [33], it was found that salt stress together with increased H_2_O_2_ induced programmed cell death increased fluorescent signals in root tips of tobacco and *Arabidopsis.* In the present study, PDL-1 showed lower intensity signals as compared to L-4076, which is attributed to high tolerance of PDL-1 towards alkalinity (Fig. 2 i-l).

Antioxidant enzyme activities and accumulation of low molecular weight osmolytes like proline, glycine betaine, together with soluble sugars and RWC were found to be higher in the tolerant cultivar (PDL-1) as compared to the sensitive one (L-4076). Antioxidant enzymes and osmolytes provide tolerance against oxidative stress in plants and helps in balancing ions in the cells [14]. Enhanced redox processes in lentil seedlings are evidenced by increased H_2_O_2_ content and subsequent increase in peroxidase activities, due to alkalinity treatment (Fig. 1 c-f and Fig. 2 i-l). Present findings are in line with the study on biochemical response towards alkalinity stress in rice [34]. Zhang et al. [34] studied effect of alkalinity on root tips and observed marked accumulation of ROS and H_2_O_2_. Further, increased antioxidant enzyme activities such as that of SOD, catalase, peroxidase and APX resulted in reduced ROS accumulation in rice roots.

In the present study, transcriptomic data also corresponded to physio-biochemical results. For example, DEG that encoded peroxidase (DN15700_c0_g1_i1), was found to be significantly up-regulated in the combination 1 T-2 T. Peroxidase enzyme is responsible for catalysis of H_2_O_2_, oxidation of toxic reductants, biosynthesis and degradation of lignin, suberization, auxin catabolism and response to oxidative stress [35]. As reported by Zhang et al. [34], alkalinity stress causes root damage by inducing accumulation of ROS and H_2_O_2_. Thus, upregulation of peroxidase in the present study indicates neutralization of ROS generated during stress. PDL-1 showed higher activities of peroxidase enzymes viz. GPX and APX which resulted in tolerance towards alkalinity stress conditions. DEGs annotated for peroxidase family (DN15700_c0_g1_i1, DN10681_c0_g1_i1) were found to be up-regulated more significantly in PDL-1 compared to L-4076. Similar results were also found in other studies in wild jujube and salsa leaves under alkalinity and salinity stress, respectively [18, 36].

### DEGs associated with cytological changes under alkalinity stress

Cells in root tips divide constantly when roots are directly exposed to high salt concentrations. Mitotic activity evaluated as MI is an important parameter for studying such cytotoxic effects. Exposure to high alkalinity stress has led to abnormal chromosomal behaviour in root tip cells of rye and other crops [37, 38]. A high amount of salt can interfere with the osmotic balance of the cells and thus can generate ROS which affects DNA [39]. Evidently, highly reactive hydroxyl radical (^•^OH) species adds double bonds to DNA bases and abstracts H atom from the thymine’s methyl group and each of the C‐H bonds of 2′‐deoxyribose [40] and therefore, have genotoxic effects on cells under salt / alkalinity stress conditions. In the present study, chromosomal aberrations and poorly dividing cells were quite visible after 24 h and 48 h of alkalinity treatment in both the cultivars. The possible explanation for the above-mentioned observation could be increase in ROS-related cytotoxicity, since high salt/alkali stress generates ROS which impacts the chromosome arrangement or can create lesions in chromosome, disrupts spindle formation, etc. [39].

Differential gene expression analysis of both the cultivars suggested involvement of genes coding for protein MEI2-like 2 (DN26324_c1_g1_i3) and serine/threonine-protein kinase fray2 (DN28256_c1_g2_i10) which are involved in regulation of cell division. The gene MEI2-like 2 is believed to be involved in meiotic cell cycle and positive regulation of meiotic nuclear division whereas, gene serine/threonine-protein kinase is involved in regulation of mitotic cell cycle, signal transduction by protein phosphorylation and in stress-activated protein kinase signalling cascade [41]. Up-regulation of such genes denotes the active response of plant cells towards alkalinity stress.

### Alkalinity responsive and tolerance genes

In order to study the molecular mechanism of alkalinity tolerance, alkalinity responsive and tolerance genes were identified by studying their differential expression in sensitive v/s tolerant cultivars under control and stress conditions. Common genes that were differentially regulated between the two cultivars under treated conditions but remained unchanged under control condition are herein mentioned as “responsive genes” while genes that were differentially regulated under stress condition in tolerant cultivar as compared to sensitive one are addressed as “tolerance genes”. Putative alkali responsive genes observed in this study have previously been reported to modulate similar or other abiotic stress responses. For example, major latex protein (MLP)-like protein has been identified as positive regulator of drought stress in *Arabidopsis* via ABA dependent pathway [42]. Similarly, Monks et al. [43] demonstrated the role of *Ssh1p*, a phosphatidylinositol transfer protein in synthesis of osmoprotectant phosphoinositide under hyper-osmotic stress. Also, Zhang et al. [34] reported enhanced activity of SOD along with accumulation of superoxide anions in roots of *Oryza sativa* during alkalinity stress*.*

Amino acid proline is amongst the most abundantly accumulated osmo-protectant in plants during stress period [44]. Similar results were found in the present investigation where, PDL-1 accumulated more proline and showed less salt injury as compared to L-4076. This is due to upregulation of Δ1-Pyrroline-5-carboxylate synthetase (P5CS), which is a rate-limiting enzyme involved in biosynthesis of proline [45]. Similar to our observation, Kishor et al. [46] observed that, over-expression of P5CS leads to osmotic tolerance induction in plants via enhanced proline production. Apart from previously reported genes, we also observed some novel alkali responsive genes such as Phytochromobilin:ferredoxin oxidoreductase, Cyclin-U1-1, MLP-like protein 423, etc. which are involved in chloroplast-nucleus signalling pathway, phytochromobilin biosynthetic process, cell division and positive regulation of ABA signal transduction, respectively. The genes involved in cell division regulation and ABA signalling are actively involved in stress tolerance mechanisms of plants [47].

In the present study, DEGs were categorized into 14 functional categories i.e. phyto-hormones, light reactions, transcription factors, stomatal regulation, mitosis, nodulation, root-shoot growth & development, epigenetics, chlorophyll synthesis, ion transporters, endo-membrane trafficking, calcium and lipid metabolism. Li et al. [19] also reported up-regulation of similar DEGs viz. light reactions, ion transport and root meristem growth in two contrasting rice genotypes under alkali stress. In another study, transcription factors like bHLH, bZIP, NAC, etc. were found to be activated along with calcium signalling induced accumulation of H_2_O_2_ and secondary metabolites in wild jujube, which is in line with this study [18].

### Alkali responsive pathways activated during stress treatment

In the present study, most of the DEGs belonged to biological and cellular processes especially to phyto-hormones and secondary metabolism involved in detoxification of alkali salts. Similar cascade of cellular response under NaHCO_3_ stress was also noticed in soybean, where alkaline modulated genes were found to be involved in metabolism, energy, signal transduction, and transcription [48]. Present findings are also in line with other studies under salinity stress [49, 50]. MapMan analyses revealed pathways which were actively involved in alkalinity response viz. secondary metabolism pathways involved in biosynthesis of flavonoids & lignin; shikimate and MVA pathways together with pathways involved in synthesis of antioxidants like phenolic acids, anthocyanins, etc. Similar to our study, Zhang et al*.* [17] also reported involvement of secondary metabolism pathways viz. phenylpropanoid and phenyl alanine metabolism in tolerance towards alkalinity stress in soybean.

Secondary metabolites such as phenolics and flavonoids are antioxidants that are involved in neutralization of ROS generated during stress [51]. These are actively recruited in tolerance mechanisms against oxidative stress [52]. Therefore, upregulation of such DEGs is well justified. Apart from secondary metabolism, phyto-hormone biosynthesis pathways, anti-oxidative enzymes, biotic and abiotic stress signalling pathways got activated during alkali treatment in both the cultivars. Similarly, other studies also found secondary metabolism, calcium signalling and phyto-hormone synthesis pathways activated during alkalinity stress conditions [18–20].

Transcription factors also play a major role in gene expression under varied environmental conditions. Major transcription factors found in response to alkalinity stress belonged to NAC (Trinity ID- DN22008_c0_g1_i2), bZIP (Trinity ID-DN14140_c0_g1_i3), and ZF (Trinity ID-DN32866_c1_g1_i6) families. Most of these transcription factors are involved in biosynthesis of chlorophyll and have regulatory function during stress response. Similar transcription factors (NAC and bZIP) were also found by Li et al. [19] in transcriptome analysis of two contrasting rice genotypes under alkali stress. Transcription factors involved in regulation of phyto-hormones i.e. ABA, jasmonates, cytokinins, indole acetic acid, etc. were also stimulated in both the cultivars under alkali stress.

During alkali stress, root cells are mostly prone to cell wall damage, which is mostly attributed to ROS generation [36]. Therefore, cultivar L-4076 showed maximum root cell damage compared to PDL-1. Activation of cell wall biosynthesis and modification mechanism is representative of cellular response against alkali stress. Evidently, major up-regulated DEGs such as Transcriptional corepressor LEUNIG_HOMOLOG (DN28191_c0_g1_i 13), Outer membrane protein Omp38 (DN12719_c0_g1_i1), Probable galacturonosyl transferase 11 (DN32815_c0_g1_i1) identified in the present study belonged to cell wall modification functional group.

### ABA signalling pathway: the most responsive pathway under alkalinity stress

ABA signalling was found to be the most prominent pathway activated in both the cultivars during alkalinity stress. Plant cells respond to various abiotic stresses in two ways; either through ABA dependent or independent pathways [53]. ABA dependent pathway is reported to be the most common pathway responsible for providing tolerance against various abiotic stresses like drought, cold, salinity etc. [54]. DEGs up-regulated between sensitive and tolerant cultivars were responsible for ABA stress signalling. Related DEGs which were significantly up-regulated in the present study includes dehydrin 1 (DHN1) (DN27872_c0_g1_i2), 9-cis-epoxycarotenoid dioxygenase 1 (NCED1) (DN29751_c0_g1_i1), ABA-responsive protein 18 (ABR18) (DN28897_c0_g1_i1) and BEL1-like homeodomain protein 1 (BLH1) (DN14830_c0_g1_i3).

DHN1 is a stress responsive protein involved in response to ABA and water scarcity [55]. Dehydrins play important role in sustaining integrity of membrane enzymes and nucleotides [56]. Dehydrin gene was also found to be up-regulated in response to drought and cold stress in transcriptome analyses of barley [57]. Similarly, Kumar et al. [58] overexpressed *OsDhn1* gene and improved drought and salinity tolerance in rice. Other genes like NCED1, ABR18 and BEL1-like homeodomain protein 1 are also involved in regulation of stress response through ABA signalling [59]. NCED catalyzes an oxidative cleavage reaction, which is the regulatory step in ABA synthesis from carotenoids [60]. On the other hand, BLH1 has role in ABA mediated seed dormancy and early seedling development [61]. In this study Kim et al. [61] found that *Arabidopsis* BLH1 over-expressing lines were hypersensitive to ABA and salinity, and exhibited increased expression of ABA-responsive genes, such as ABI3 and ABI5. Up-regulation of ABA responsive DEGs in the present investigation clearly indicates the role of ABA hormone in response to the alkalinity stress in both the cultivars. On the contrary, ABR18-like genes were found to be downregulated in tolerant chickpea cultivars as compared to the sensitive ones under salt stress [62].

## Identification of molecular markers

SSRs and SNPs are considered to be invaluable markers for crop breeding. Under control conditions, both the cultivars showed maximum variants compared to the treated conditions. The data generated in this study will be helpful in developing an understanding of molecular response of plants under alkalinity stress which can be utilized in future breeding programs of lentil and related species.

## qRT-PCR validation

Ten DEGs selected for qRT-PCR validation were involved in ABA signal transduction; hormones and ion transport; proline accumulation and other regulatory genes. For 1C-1 T, three genes were used: proline dehydrogenase 2, MLP-like protein 423, and cyclin-dependent kinase G-2 (CDKG-2). Proline dehydrogenase is responsible for oxidation of proline and synthesis of glutamate. Its suppression causes proline accumulation during abiotic stresses and thus enhances resistance towards these stresses [63]. MLP-like protein 23 gene is involved in ABA response regulation in *Arabidopsis* [40]. CDKG-2 is involved in cell cycle and gene expression regulation and has an important role in regulation of cellular processes during abiotic stresses [64].

For 2C-2 T, three genes namely, inactive poly [ADP-ribose] polymerase RCD1, xyloglucan endotransglucosylase and ferritin-2 were employed. Inactive poly [ADP-ribose] polymerase RCD1 gene is involved in regulation of oxidative stress, hormonal and developmental response, etc. [64]. Gene xyloglucan endotransglucosylase is involved in abiotic stress response and participate in many physiological roles such as cell wall modifications during stress [65]. The gene was found to be responsible for improved drought and salt tolerance in transgenic *Arabidopsis* [66]. Ferritin gene is involved in abiotic stress response in plants and in ROS scavenging activities. In a study on wheat, over expression of ferritin gene conferred improved tolerance to heat and other abiotic stresses [67].

For 1 T-2 T, four genes namely, auxin transport protein BIG, ABC transporter G family member 25, protein ZINC INDUCED FACILITATOR-LIKE 1 and probable serine/threonine-protein kinase SIS8 were used for validation of identified DEGs. Most of the genes used were involved in abiotic stress response, transport of ion and, hormones, etc.

BIG is required for auxin efflux and polar auxin transport which affects auxin mediated developmental responses like cell elongation, lateral root development, apical dominance etc. [68]. ABC transporter G family member 25 arbitrates ABA export through plasma membrane, especially in vascular tissues and brings in intercellular ABA signalling [69]. ZINC INDUCED FACILITATOR-LIKE 1 is associated with auxin efflux and functions as a positive regulator of upward transport towards shoot at the root apex. It also imparts proton efflux from the vacuolar compartment [70]. Probable serine/threonine-protein kinase SIS8 has been reported to act as a negative regulator of salt tolerance in *Arabidopsis* [71]. Conclusively, the qRT-PCR expression patterns validated differential expression of genes obtained from Illumina sequencing.

## Conclusions

Present investigation reports the transcriptome analysis of lentil seedlings and its putative molecular mechanism in response to alkalinity stress. The study revealed that active pathways for alkalinity stress tolerance in lentil are phyto-hormones biosynthesis—predominantly through ABA signalling and secondary metabolism, which are also known to be actively involved in other abiotic stress response mechanisms. PDL-1 showed pronounced tolerance towards alkali stress compared to L-4076 owing to low accumulation of Na^+^ and high accumulation of K^+^ in both roots and shoots. The cultivar also maintained high cellular integrity and physiol-cytological status under stress condition based on mitotic index, antioxidant production, osmolytes accumulation and membrane stability. Since, the cultivar was previously found to be resistant to drought and salinity also; it can be utilized as novel genetic resource for climate smart lentil breeding. SSRs and SNPs identified under alkali stress response can be developed as genomic resources for mapping and tagging of genes for breeding alkali tolerance traits in lentils and related species.

## Material and method

### Plant material, growth and alkalinity treatment

Two cultivars namely, PDL-1 (alkalinity tolerant) and L-4076 (alkalinity sensitive) were assayed to study the effects of alkalinity stress on plants. These two cultivars were developed at Indian Council of Agricultural Research (ICAR)-Indian Agricultural Research Institute (IARI), New Delhi, India. The experiments were conducted under hydroponic condition provided at National Phytotron Facility (NPF), ICAR-IARI, New Delhi, India. Sterilized seeds of both the cultivars were germinated under control condition, programmed at a temperature of 20–24 °C for 16 h light/8 h dark photoperiod.

### Characterization of morpho-anatomical and cytological changes

Seven days old seedlings were transferred to hydroponics, wherein they were subjected to alkalinity stress (40 mM NaHCO_3_; pH 9.1) conditions for next 3, 5 and 7 days. No trace of NaHCO_3_ was added to control._._The chemical composition of the nutrient solution and treatment conditions were applied following the methods described by Ge et al. [48] with partial modifications. Plants of tolerant cultivar (PDL-1) under control condition were named 1C followed by sensitive cultivar (L-4076) under control condition as 2C. Similarly, tolerant and sensitive cultivars when exposed to 40 mM NaHCO_3_ stress were named as 1 T and 2 T, respectively. To examine the effects of alkalinity stress on morphological traits at different time lengths, the most prominent symptoms i.e. wilting and necrosis were evaluated.

Anatomical changes within the contrasting cultivars were checked by free hand cross sectioning of roots and shoots using razor blades. Toluidine blue (50%) staining method was applied for categorization of tissues on the basis of different colours. These cross-sections were visualised as well as photographed under optical microscope (Zeiss AXIOSKOP 2, Germany).

Effects of alkalinity stress on roots were studied by calculating MI in root tips after 4 h, 6 h, 24 h and 48 h of treatment. Root tips from five seedlings were taken and stained as described by Zhang et al. [72]. MI was calculated by dividing average number of dividing cells with average number of cells in the five microscopic field views under observation at a particular time.

### Evaluation of physio-biochemical parameters

To differentiate physio-biochemical activities within the leaves of tolerant and sensitive cultivars under control and alkalinity stress conditions, parameters such as RWC, MSI and photosynthetic pigments (total chlorophyll) were measured. Antioxidants enzymes such as SOD, APX, GPX and catalase were also deduced. Accumulation of different osmolytes such as glycine betaine, proline and soluble sugars were estimated for both the cultivars. All the above-mentioned parameters were deduced following the methods described by Singh et al. [15, 22, 73]. ABA was also estimated from the roots of both the cultivars under control and stress conditions following the protocol described by Zeevaart [74]. Na^+^ and K^+^ contents in the roots and leaves were elucidated under control and alkalinity stress conditions using Flame Photometer (Systronics, India) as described by Singh et al*.* [4]. H_2_O_2_ levels in root tips were detected using FDA staining method following Singh et al. [4].

### RNA isolation and Complementary Deoxy-ribonucleic acid (cDNA) library preparation

The transcriptomic experiment was conducted using three biological replicates, consisting of 12 seedlings each from the contrasting cultivars (PDL-1 and L-4076) under control and alkalinity stress conditions. Total RNA was isolated from seedling leaf tissue, using QIAGEN RNeasy Plant Mini kit (QIAGEN, Hilden, Germany). Isolated RNA was purified and checked for integrity using Bioanalyzer (Agilent 2100, USA) to follow Illumina library construction and qRT-PCR. From the total extracted RNA, poly(A) messenger RNA (mRNA) was isolated using oligo(dT) beads-based affinity chromatography. Library was prepared using TruSeq™ RNA Sample Prep kit (Illumina Inc., USA). Isolated and enriched mRNA was further fragmented using magnetic beads containing poly(T) molecules, which was then reverse transcribed into cDNA using random primers. The quality and quantity of cDNA was assessed using Agilent 2100 bioanalyzer and cDNA was purified using AMPure XP beads (Beckman Coulter, USA). Adapters were ligated to the ends of repaired cDNA fragments. Clusters were generated by selective amplification of fragments which have adapters ligated at both the ends. Sequencing was performed on Illumina HiSeq 2000 platform (Illumina, Inc. USA) to generate 2 × 100 bp paired end reads.

### Sequencing, data filtering and assembly

Raw data obtained from sequencing was converted into nucleotide sequence by base-calling and selective filtration to remove reads below 30 bp and having Phred quality score (Q) less than 20. Prior to de novo transcriptome assembly, raw Fastq files were trimmed by removing the first two bases, last ten bases and adapter sequences from all the reads. Quality filtered reads thus generated were taken further for de novo transcriptome assembly using de novo Assembler (Trinity V 2.8.6) [75]. De novo contigs were obtained by assembling clean paired end reads. Trimmed reads were aligned to the assembled transcriptome using Bowtie program (V 1.2.3) [76].

### Differential gene expression and functional annotation

DEGs were identified using EdgeR software with set parameters including read counts ≥ 1, *p* adjusted ≤ 0.05, false discovery rate (FDR) < 0.01 and absolute log_2_fold change ≥ 1. The transcripts that were differentially expressed were annotated by basic local alignment search tool (BLAST) of National Centre for Biotechnology Information (NCBI) with non-redundant (nr) protein database. Only the matches with E-value ≤ 10^–5^ as well as similarity score ≥ 40% were further used for functional annotation. Gene Ontology (GO) terms of transcripts were extracted for annotated DEGs and then segregated into different functional categories using Web Gene Ontology Annotation (WEGO V 2.0) [77]. Mapman (v3.51R2) analysis was performed to associate molecular pathways with annotated DEGs [78].

### SNP and SSR calling

To further utilize the application of transcriptomic data, SNP and SSR variants were called from the assembled RNA-Seq data. SAMtools mpileup (V2.1) [79] and custom script were used for SNP variant calling based on the minimum read depth of 10. Genome Analysis Tool Kit (GATK) [80] software was used for calling SNPs using haplotype caller (command version 3.6–0) with all the default parameters. For SSR calling, high quality filtered reads that aligned with the contigs were deduced using MIcro SAtellite identification (MISA, V1.1) software [81].

### qRT-PCR validation

For qRT-PCR validation, 10 significant genes were organized in three different combinations for comparison of expression between 1C and 1 T; 1 T and 2 T; 2C and 2 T. Total RNA was isolated from the leaves of control and alkalinity-treated cultivars (PDL-1 and L-4076). RNA was quantified using NanoDrop™ Spectrophotometer (Thermo Fisher Scientific, USA) and reverse transcribed to cDNA using Biorad cDNA synthesis kit (Biorad, USA). The expression of genes was normalized using β-tubulin as the reference gene. Primers were designed using Primer3Plus software. The qRT-PCR reaction was carried out in 25 μL PCR reaction mixture consisting of 4 μg diluted cDNA, 4 μL each of forward and reverse primers and 12 μL SYBR Green dye in qRT-PCR cycler (Bio-Rad, USA) programmed at the following PCR cycle: 50 °C for 2 min, 95 °C for 10 min and 40 cycles consisting of 95 °C for 15 s and 60 °C for 1 min. Relative quantification of target genes expression was calculated using 2^−ΔΔCt^ method [82].

## Supplementary Information

**Additional file 1: Fig.S1.** Circos representing distribution of differentially expressed genes (DEGs) in lentil cultivars between combinations a) 1T-1C b) 2T-2C and 1T-2T, where 1C: PDL-1 control, 1T: PDL-1 treated, 2C: L-4076 control and 2T: L-4602 treated.

**Additional file 2: Fig. S2.** Relative expression of differentially expressed genes (DEGs) related to different cytological processes under alkalinity stress in lentil cultivars represented through BINs and Sub-BINs using MapMan Software.

**Additional file 3: Fig. S3.** Regression graph between expression data of next generation sequencing (NGS) and quantitative real time-polymerase chain reaction (qRT-PCR) of lentil cultivars under alkalinity stress.

**Additional file 4: Table S1.** Changes in mitotic index (MI) of lentil cultivars under normal and alkalinity treated conditions.

**Additional file 5: Table S2.** Details of contigs generated through *de novo* assembly in lentil samples under alkalinity stress.

**Additional file 6: Table S3.** Alkalinity responsive genes with log_2_fold changes above 3 under alkalinity stress conditions in two contrasting lentil cultivars.

**Additional file 7: Table S4.** Categorical representation of different differentially expressed genes (DEGs) involved in alkalinity stress response in lentil cultivars.

**Additional file 8: Table S5.** List of 10 primers from combinations 1C-1T, 2C-2T and 1T-2T used for validation of next-generation sequencing (NGS) data generated from lentil cultivars under alkalinity stress, where 1C: PDL-1 control, 1T: PDL-1 treated, 2C: L-4076 control and 2T: L-4602 treated.

## Data Availability

All the data generated or analyzed during this study are included in this manuscript and its supporting information files. Sequences were deposited in Sequence Read Archive (SRA) (BioProject Accession: PRJNA685293).
