# Supplementary figures and images for: Linking genome wide RNA sequencing with physio-biochemical and cytological responses to catalogue key genes and metabolic pathways for alkalinity stress tolerance in lentil (Lens culinaris Medikus)
Source: BMC Plant Biol. 2022 Mar 5;22:99. doi: 10.1186/s12870-022-03489-w (PMC8897830; doi:10.1186/s12870-022-03489-w)

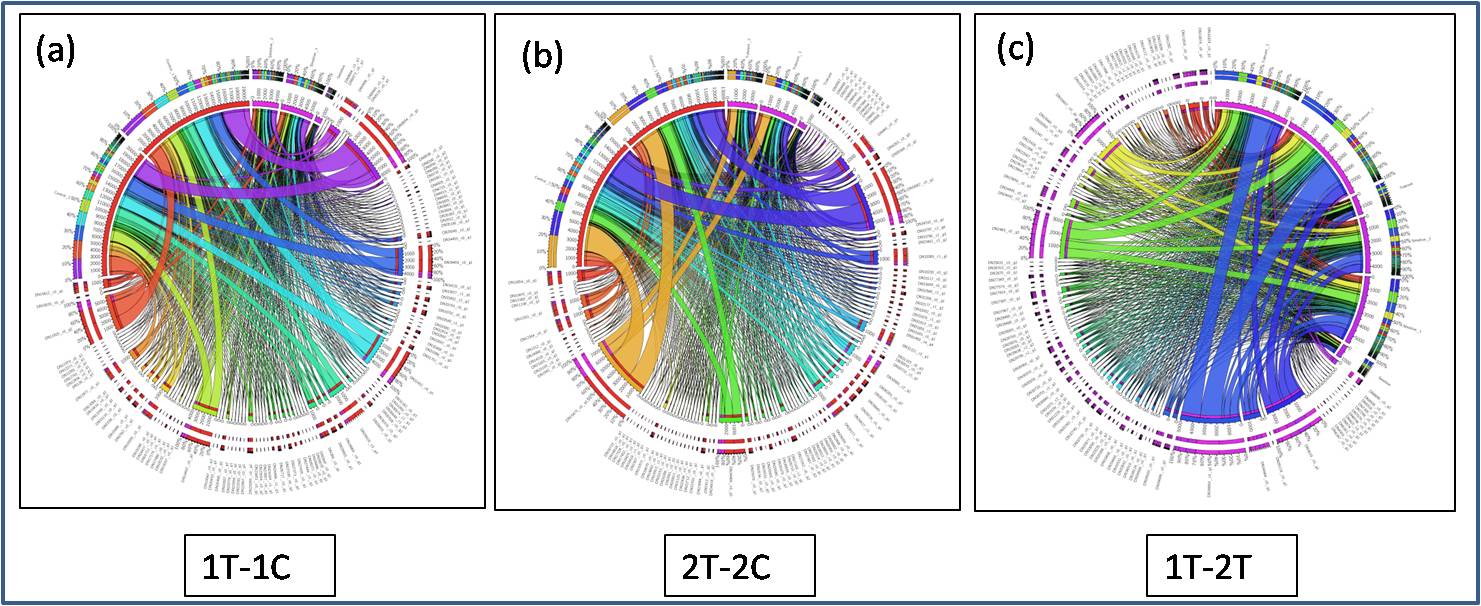

Supplement: Supplementary file 1 — Additional file 1: Fig.S1. Circos representing distribution of differentially expressed genes (DEGs) in lentil cultivars between combinations a) 1T-1C b) 2T-2C and 1T-2T, where 1C: PDL-1 control, 1T: PDL-1 treated, 2C: L-4076 control and 2T: L-4602 treated. [file 12870_2022_3489_MOESM1_ESM.jpg]

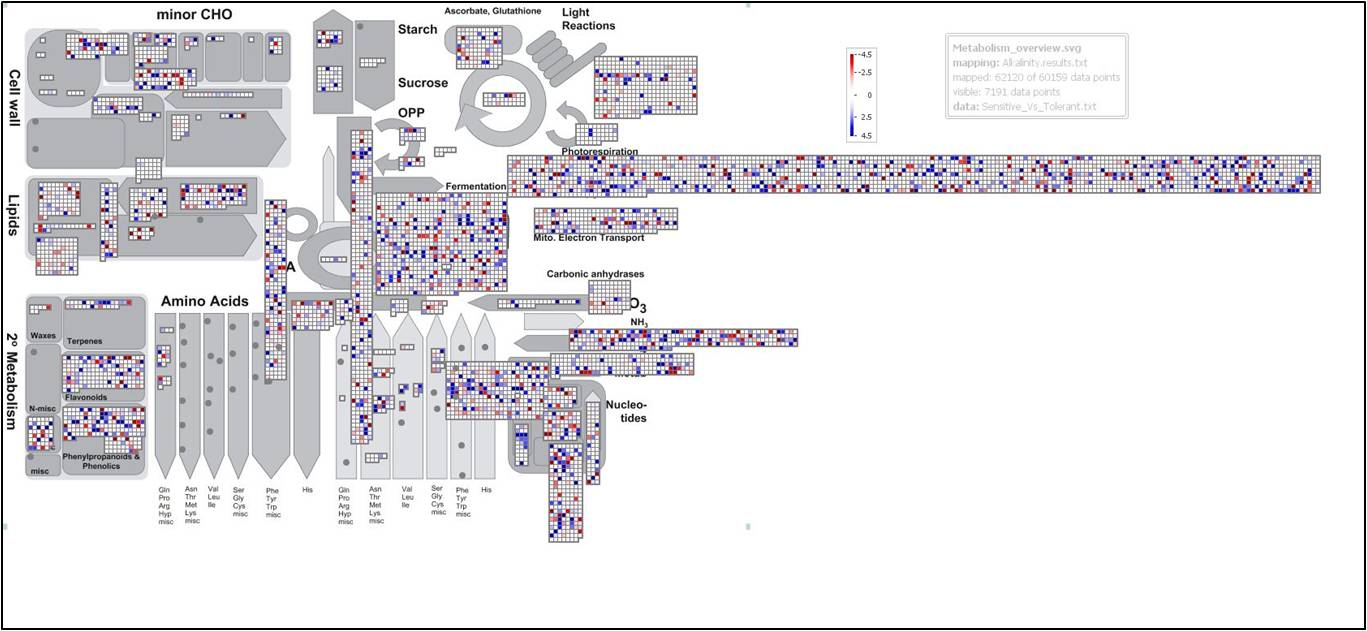

Supplement: Supplementary file 2 — Additional file 2: Fig. S2. Relative expression of differentially expressed genes (DEGs) related to different cytological processes under alkalinity stress in lentil cultivars represented through BINs and Sub-BINs using MapMan Software. [file 12870_2022_3489_MOESM2_ESM.jpg]

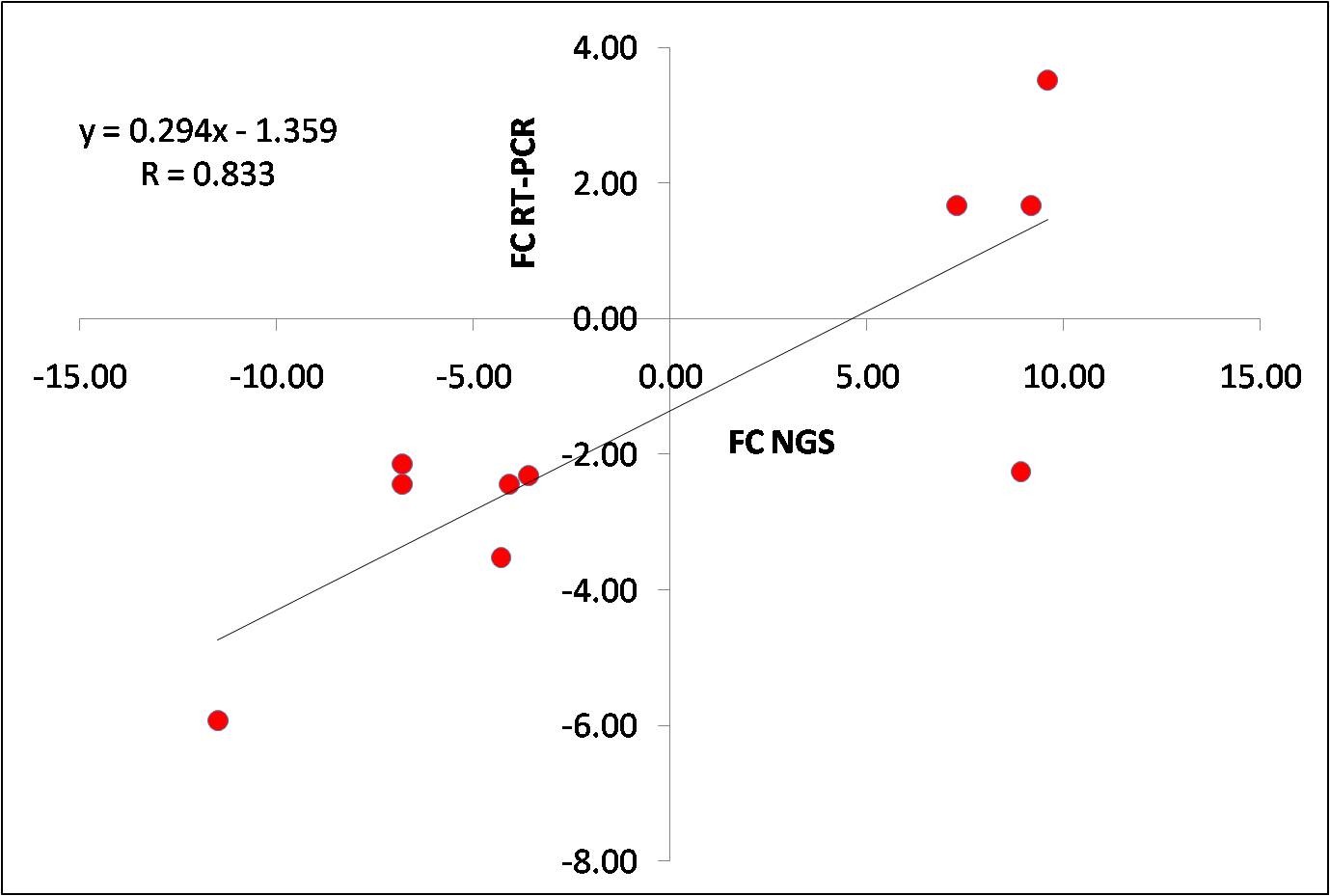

Supplement: Supplementary file 3 — Additional file 3: Fig. S3. Regression graph between expression data of next generation sequencing (NGS) and quantitative real time-polymerase chain reaction (qRT-PCR) of lentil cultivars under alkalinity stress. [file 12870_2022_3489_MOESM3_ESM.jpg]
