# Supplementary material for: Linking genome wide RNA sequencing with physio-biochemical and cytological responses to catalogue key genes and metabolic pathways for alkalinity stress tolerance in lentil (Lens culinaris Medikus)
Source: BMC Plant Biol. 2022 Mar 5;22:99. doi: 10.1186/s12870-022-03489-w (PMC8897830; doi:10.1186/s12870-022-03489-w)
Supplement: Supplementary file 4 — Additional file 4: Table S1. Changes in mitotic index (MI) of lentil cultivars under normal and alkalinity treated conditions. [file 12870_2022_3489_MOESM4_ESM.docx]

Supp Table 1: Changes in mitotic index (MI) of lentil cultivars under normal and after 6 h of alkalinity treated conditions.

| S. No. | Parameter | 1C | 1 T | 2C | 2T |
| --- | --- | --- | --- | --- | --- |
| 1 | Average cells in the field view | 50 | 47 | 53 | 67 |
| 2 | Average no. of dividing cells | 10 | 7 | 12 | 6 |
| 3 | Average no. of aberrantly dividing cells | 0 | 3 | 0 | 6 |
| 4 | Mitotic index (%) | 20 | 14.8 | 22.6 | 8.9 |
| 1C: tolerant cultivar, PDL-1 under control condition; 1T: PDL-1 under alkalinity stress condition; 2C: sensitive cultivar, L-4076 under control condition; 2T: L-4076 under alkalinity stress condition. | | | | | |
