# Supplementary material for: Linking genome wide RNA sequencing with physio-biochemical and cytological responses to catalogue key genes and metabolic pathways for alkalinity stress tolerance in lentil (Lens culinaris Medikus)
Source: BMC Plant Biol. 2022 Mar 5;22:99. doi: 10.1186/s12870-022-03489-w (PMC8897830; doi:10.1186/s12870-022-03489-w)
Supplement: Supplementary file 6 — Additional file 6: Table S3. Alkalinity responsive genes with log2fold changes above 3 under alkalinity stress conditions in two contrasting lentil cultivars. [file 12870_2022_3489_MOESM6_ESM.docx]

Supp Table S3: Alkalinity responsive genes with log_2_fold changes above 3 under stress conditions in two cultivars

| Gene id | Gene description | Gene function |
| --- | --- | --- |
| DN19992_c0_g1_i4 | MLP-like protein 423; | Positive regulation of ABA and  Signalling transduction |
| DN30347_c0_g1_i7 | Lysine-specific histone demethylase 1 homolog 3 | Chromatin Organization |
| DN18507_c0_g1_i2 | Cytochrome c oxidase subunit 5b-2, mitochondrial | Mitochondrial ATP synthesis coupled  proton transport |
| DN33607_c0_g1_i9 | Protein PHYLLO, chloroplastic | Multifunctional enzyme required  for phylloquinone (vitamin K1)  biosynthesis |
| DN30052_c0_g3_i1 | Glutamate dehydrogenase 2 | Involved in glutamate biosynthetic process |
| DN28224_c2_g2_i9 | Phosphatidylinositol/phosphatidylcholine transfer protein SFH9 | Regulates protein transport, root epidermal cell differentiation, root hair cell tip growth and root hair elongation |
| DN28821_c2_g1_i4 | Kinesin-like protein KIN-4A | It drives long-distance transport of  cargo along cortical microtubules and contributes to cortical microtubule  mediated trafficking of cell wall  components |
| DN33323_c0_g3_i4 | Nuclear pore complex protein NUP205 | mRNA and protein transport |
| DN26708_c0_g1_i4 | Superoxide dismutase [Fe] 3, chloroplastic | Metal ion binding |
| DN32138_c1_g1_i17 | Autophagy-related protein 9 | Involved in autophagy and cytoplasm  to vacuole transport (Cvt) vesicle  formation |
| DN25677_c0_g1_i2 | Probable phospholipid-transporting ATPase 11 | ATPase-coupled intra membrane lipid  transporter activity |
| DN29643_c0_g1_i17 | Folylpolyglutamate synthase | Folic acid-containing compound biosynthetic process, nucleobase containing compound metabolic process |
| DN32070_c0_g2_i13 | Protein CHROMATIN REMODELING 5 | Chromatin organization |
| DN32676_c0_g1_i4 | Exportin-7-B | Protein export from nucleus |
| DN33106_c0_g1_i3 | Bidirectional sugar transporter SWEET2 | Carbohydrate transport |
| DN31046_c0_g1_i1 | RING-H2 finger protein ATL66 | Cellular response to hypoxia,  protein ubiquitination |
| DN31103_c0_g2_i5 | Protein ZINC INDUCED FACILITATOR-LIKE 1 | Basipetal auxin transport, regulation of stomatal closure, response to water deprivation and root development |
| DN32489_c0_g1_i1 | DNA ligase 1 | It seals nicks in double-stranded DNA  during DNA replication, DNA  recombination and  DNA repair |
| DN30223_c0_g1_i2 | Callose synthase 9 | Cell wall reinforcement |
| DN28524_c0_g1_i1 | Cell division control protein 48 homolog C | Probably functions in cell division, homotypic vesicular fusion and growth processes |
| DN33446_c0_g1_i4 | Protein WHAT'S THIS FACTOR 1 homolog, chloroplastic | Group II intron splicing and photosynthesis |
| DN28805_c0_g1_i2 | Eugenol synthase 1 | Phenylpropanoid biosynthetic process |
| DN33715_c0_g1_i1 | Endoribonuclease Dicer homolog 3a | Involved in the RNA silencing pathway |
| DN30763_c0_g2_i7 | Delta-1-pyrroline-5-carboxylate synthase | Bifunctional enzyme that converts glutamate to glutamate 5-semialdehyde, an intermediate in the biosynthesis of proline, ornithine and arginine |
| DN31127_c0_g1_i10 | Protein phosphatase 2C and cyclic nucleotide-binding/kinase domain-containing protein | Signal transduction |
| DN32544_c0_g1_i3 | WAT1-related protein At3g28050 | Transmembrane transporter activity |
| DN26098_c1_g1_i3 | Cyclin-U1-1 | Cell division, regulation of cyclin  dependent protein serine/threonine  kinase activity |
| DN26947_c0_g1_i1 | Phytochromobilin:ferredoxin oxidoreductase, chloroplastic | Chloroplast-nucleus signaling pathway,  phytochromobilin biosynthetic  process |
| DN29941_c0_g1_i6 | Lupeol synthase | Cuticle development, pentacyclic triterpenoid biosynthetic process |
