# Supplementary material for: Linking genome wide RNA sequencing with physio-biochemical and cytological responses to catalogue key genes and metabolic pathways for alkalinity stress tolerance in lentil (Lens culinaris Medikus)
Source: BMC Plant Biol. 2022 Mar 5;22:99. doi: 10.1186/s12870-022-03489-w (PMC8897830; doi:10.1186/s12870-022-03489-w)
Supplement: Supplementary file 7 — Additional file 7: Table S4. Categorical representation of different differentially expressed genes (DEGs) involved in alkalinity stress response in lentil cultivars. [file 12870_2022_3489_MOESM7_ESM.docx]

Supp Table 4: Categorical representation of different DEGs involved in alkalinity stress response

| Gene Id | Gene annotation | Gene description | log_2_Fold  Change | Gene function |
| --- | --- | --- | --- | --- |
| DN28146_c0_g2_i1 | SLAH3_ARATH | S-type anion channel SLAH3 | 6.59 | Stomatal regulation |
| DN29037_c1_g1_i10 | AB5C_ARATH | ABC transporter C family member 5 | 6.19 |  |
| DN21509_c2_g2_i1 | JA2_SOLLC | NAC domain-containing protein JA2 | 2.61 |  |
| DN31494_c0_g1_i1 | PHOT1_ARATH | Phototropin-1 | -5.97 |  |
| DN24304_c0_g1_i1 | ZIFL1_ARATH | Protein ZINC INDUCED FACILITATOR-LIKE 1 | -6.80 |  |
| DN33722_c0_g1_i2 | BIG_ARATH | Auxin transport protein BIG | 9.61 | Root-Shoot growth and development |
| DN33322_c0_g1_i5 | AB20B_ARATH | ABC transporter B family member 20 | 6.19 |  |
| DN24953_c0_g1_i1 | PTR16_ARATH | Protein NRT1/ PTR FAMILY 4.3 | 6.81 |  |
| DN29037_c1_g1_i10 | AB5C_ARATH | ABC transporter C family member 5 | 6.19 |  |
| DN33414_c0_g1_i2 | MAIL2_ARATH | Protein MAIN-LIKE 2 | 6.03 |  |
| DN30304_c1_g1_i12 | CD27B_ARATH | Cell division cycle protein 27 homolog B | 5.10 |  |
| DN30763_c0_g2_i7 | P5CS_MESCR | Delta-1-pyrroline-5-carboxylate synthase | 4.51 |  |
| DN21754_c0_g1_i1 | PCP13_ARATH | Precursor of CEP13 | 4.37 |  |
| DN24304_c0_g1_i1 | ZIFL1_ARATH | Protein ZINC INDUCED FACILITATOR-LIKE 1 | -6.80 |  |
| DN21327_c0_g1_i4 | CIP73_LOTJA | Ubiquitin-like domain-containing protein CIP73 | 6.38 | Nodulation |
| DN30084_c0_g1_i2 | LYK3_MEDTR | LysM domain receptor-like kinase 3 | 5.83 |  |
| DN33463_c0_g1_i8 | LIN_MEDTR | Putative E3 ubiquitin-protein ligase LIN | 5.49 |  |
| DN30674_c3_g2_i3 | CHLH_ARATH | Magnesium-chelatase subunit ChlH, chloroplastic | -5.13 |  |
| DN33463_c0_g1_i6 | LIN_MEDTR | Putative E3 ubiquitin-protein ligase LIN | -6.01 |  |
| DN21124_c0_g1_i4 | CIP73_LOTJA | Ubiquitin-like domain-containing protein CIP73 | -6.26 |  |
| DN30920_c1_g6_i3 | PUB44_ARATH | U-box domain-containing protein 44 | 7.56 | Chlorophyll Synthesis |
| DN30112_c0_g1_i10 | PIF1_ARATH | Transcription factor PIF1 | 6.11 |  |
| DN31657_c1_g1_i13 | Y1457_ARATH | Acyltransferase-like protein At1g54570, chloroplastic | 5.91 |  |
| DN31657_c1_g1_i5 | Y1457_ARATH | Acyltransferase-like protein At1g54570, chloroplastic | 5.88 |  |
| DN32913_c0_g1_i1 | HEM11_CUCSA | Glutamyl-tRna reductase 1, chloroplastic | 4.97 |  |
| DN30977_c0_g1_i7 | EFNMT_HUMAN | eEF1A lysine and N-terminal methyltransferase | 7.11 | Epigenetics |
| DN28191_c0_g1_i13 | LUH_ARATH | Transcriptional corepressor LEUNIG_HOMOLOG | 6.93 |  |
| DN32402_c1_g2_i7 | MOM1_ARATH | Helicase protein MOM1 | 6.78 |  |
| DN32402_c1_g2_i7 | MOM1_ARATH | Helicase protein MOM1 | 6.78 |  |
| DN32730_c0_g1_i1 | LDL3_ARATH | Lysine-specific histone demethylase 1 homolog 3 | 6.30 |  |
| DN32998_c1_g6_i2 | FGT1_ARATH | Protein FORGETTER 1 | 6.27 |  |
| DN29057_c0_g1_i2 | SFH6_ARATH | Phosphatidylinositol/phosphatidylcholine transfer protein SFH6 | 7.90 | Endomembrane trafficking |
| DN30586_c2_g3_i6 | CLASP_ARATH | CLIP-associated protein | 7.48 |  |
| DN31235_c0_g1_i3 | VPS18_ARATH | Vacuolar sorting protein 18 | 7.38 |  |
| DN25517_c0_g1_i7 | ZDH17_ARATH | Probable protein S-acyltransferase 19 | 7.17 |  |
| DN31701_c0_g1_i1 | COG5_ARATH | Conserved oligomeric Golgi complex subunit 5 | 6.89 |  |
| DN32693_c0_g1_i4 | ATG2_ARATH | Autophagy-related protein 2 | 6.78 |  |
| DN30586_c2_g3_i6 | CLASP_ARATH | CLIP-associated protein | 7.48 | Mitosis |
| DN29725_c0_g1_i4 | RIO1_DICDI | Serine/threonine-protein kinase rio1 | 7.10 |  |
| DN32392_c3_g1_i17 | TMM8B_BOVIN | Transmembrane protein 8B | 6.92 |  |
| DN13797_c0_g1_i3 | CDKG2_ORYSJ | Cyclin-dependent kinase G-2 | 6.89 |  |
| DN31928_c0_g1_i1 | FH5_ARATH | Formin-like protein 5 | 6.37 |  |
| DN24686_c0_g1_i3 | FPP_SOLLC | Filament-like plant protein | 6.30 |  |
| DN30088_c0_g1_i1 | KN14I_ORYSJ | Kinesin-like protein KIN-14I {ECO:0000305} | 6.29 |  |
| DN28401_c0_g1_i11 | ZW10_ARATH | Centromere/kinetochore protein zw10 homolog | 6.19 |  |
| DN26561_c0_g1_i4 | CCT13_ORYSJ | Cyclin-T1-3 | 6.15 |  |
| DN30920_c1_g6_i3 | PUB44_ARATH | U-box domain-containing protein 44 | 7.56 | Phyto-hormones |
| DN31703_c0_g1_i2 | AB25G_ARATH | ABC transporter G family member 25 | 7.29 |  |
| DN28412_c1_g1_i2 | PAL1_PEA | Phenylalanine ammonia-lyase 1 | 6.70 |  |
| DN20148_c0_g1_i8 | NUDT8_ARATH | Nudix hydrolase 8 | 6.36 |  |
| DN30670_c0_g1_i2 | AHK3_ARATH | Histidine kinase 3 | 7.37 |  |
| DN26864_c0_g1_i2 | PUP1_ARATH | Purine permease 1 | 3.61 |  |
| DN18075_c0_g1_i2 | RTE1_ARATH | Protein REVERSION-TO-ETHYLENE SENSITIVITY1 | 6.47 |  |
| DN29194_c1_g1_i9 | RBK1_ARATH | Receptor-like cytosolic serine/threonine-protein kinase RBK1 | 6.17 |  |
| DN33722_c0_g1_i2 | BIG_ARATH | Auxin transport protein BIG | 9.61 |  |
| DN28191_c0_g1_i13 | LUH_ARATH | Transcriptional corepressor LEUNIG_HOMOLOG | 6.93 |  |
| DN27445_c1_g1_i3 | TPR4_ARATH | Topless-related protein 4 | 6.50 |  |
| DN30112_c0_g1_i10 | PIF1_ARATH | Transcription factor PIF1 | 6.11 |  |
| DN25381_c0_g1_i1 | HSD1B_ARATH | 11-beta-hydroxysteroid dehydrogenase 1B | 6.71 |  |
| DN25025_c0_g1_i5 | IWS1_ARATH | Protein IWS1 homolog 1 | 6.27 |  |
| DN33011_c0_g2_i6 | TOR_ARATH | Serine/threonine-protein kinase TOR | 6.23 |  |
| DN30905_c0_g1_i4 | HNRPQ_ARATH | Heterogeneous nuclear ribonucleoprotein Q | 3.54 |  |
| DN32630_c1_g1_i2 | CHS4_MEDSA | Chalcone synthase 4 | 2.89 |  |
| DN11641_c0_g1_i1 | 4CLL7_ARATH | 4-coumarate--CoA ligase-like 7 | 2.22 |  |
| DN31793_c0_g1_i4 | CAMK4_ARATH | CDPK-related kinase 4 | 6.71 | Calcium and lipid signaling |
| DN19419_c0_g1_i1 | CB60A_ARATH | Calmodulin-binding protein 60 A {ECO:0000303\|PubMed:11782485} | -6.80 |  |
| DN29145_c0_g1_i2 | CIPK1_ARATH | CBL-interacting serine/threonine-protein kinase 1 | 6.11 |  |
| DN31793_c0_g1_i4 | CAMK4_ARATH | CDPK-related kinase 4 | 6.71 |  |
| DN7210_c0_g1_i1 | IMPL1_ARATH | Phosphatase IMPL1, chloroplastic | 6.14 |  |
| DN32049_c0_g1_i1 | NET1A_ARATH | Protein NETWORKED 1A | 5.54 |  |
| DN33029_c0_g2_i2 | P4KG2_ARATH | Phosphatidylinositol 4-kinase gamma 2 | -1.95 |  |
| DN14140_c0_g1_i3 | HAT5_ARATH | Homeobox-leucine zipper protein HAT5 | 6.15 | Transcription factors |
| DN32202_c0_g1_i11 | ATHB8_ARATH | Homeobox-leucine zipper protein ATHB-8 | 6.03 |  |
| DN32866_c1_g1_i1 | C3H55_ORYSJ | Zinc finger CCCH domain-containing protein 55 | 7.32 |  |
| DN32866_c1_g3_i10 | C3H38_ARATH | Zinc finger CCCH domain-containing protein 38 | 6.783128303 |  |
| DN29318_c0_g1_i3 | SRO1_ARATH | Probable inactive poly [ADP-ribose] polymerase SRO1 | 5.95 |  |
| DN30371_c0_g1_i5 | Y3037_ARATH | Probable inactive leucine-rich repeat receptor-like protein kinase At3g03770 | 5.48 |  |
| DN22008_c0_g1_i3 | .C2_ARATH | NAC domain-containing protein 2 | 4.07 |  |
| DN21509_c2_g1_i1 | JA2L_SOLLC | NAC domain-containing protein JA2L | 3.63 |  |
| DN7912_c0_g1_i1 | PMA4_NICPL | Plasma membrane ATPase 4 | 10.25 | Ion transporters |
| DN24221_c0_g1_i8 | CCC1_ARATH | Cation-chloride cotransporter 1 | 7.40 |  |
| DN28146_c0_g2_i1 | SLAH3_ARATH | S-type anion channel SLAH3 | 6.59 |  |
| DN29832_c1_g1_i3 | SUT33_ARATH | Probable sulfate transporter 3.3 | 6.41 |  |
| DN26217_c0_g1_i4 | PIF4_ARATH | Transcription factor PIF4 | 4.92 | Light responses |
| DN31545_c1_g1_i14 | DET1_SOLLC | Light-mediated development protein DET1 | 4.88 |  |
| DN23416_c0_g1_i7 | CTPA2_ARATH | Carboxyl-terminal-processing peptidase 2, chloroplastic | 4.50 |  |
| DN14830_c0_g1_i3 | BLH1_ARATH | BEL1-like homeodomain protein 1 | 4.22 |  |
| DN20691_c0_g1_i11 | KN14U_ARATH | Kinesin-like protein KIN-14U {ECO:0000305} | 5.89 | Cell wall modification |
| DN33692_c0_g1_i2 | CALS8_ARATH | Putative callose synthase 8 | 4.76 |  |
| DN25475_c0_g1_i2 | ARAE1_ARATH | UDP-arabinose 4-epimerase 1 | 3.88 |  |
| DN25941_c0_g1_i3 | RWA2_ARATH | Protein REDUCED WALL ACETYLATION 2 | 2.56 |  |
| DN30763_c0_g2_i7 | P5CS_MESCR | Delta-1-pyrroline-5-carboxylate synthase | 4.51 | Antioxidant and Oxidoreductases |
| DN23416_c0_g1_i7 | CTPA2_ARATH | Carboxyl-terminal-processing peptidase 2, chloroplastic | 4.50 |  |
| DN30523_c0_g1_i1 | C81E8_MEDTR | Cytochrome P450 81E8 | 4.14 |  |
| DN6202_c0_g1_i1 | PER2_ARAHY | Cationic peroxidase 2 | 3.96 |  |
