# Supplementary material for: Linking genome wide RNA sequencing with physio-biochemical and cytological responses to catalogue key genes and metabolic pathways for alkalinity stress tolerance in lentil (Lens culinaris Medikus)
Source: BMC Plant Biol. 2022 Mar 5;22:99. doi: 10.1186/s12870-022-03489-w (PMC8897830; doi:10.1186/s12870-022-03489-w)
Supplement: Supplementary file 8 — Additional file 8: Table S5. List of 10 primers from combinations 1C-1T, 2C-2T and 1T-2T used for validation of next-generation sequencing (NGS) data generated from lentil cultivars under alkalinity stress, where 1C: PDL-1 control, 1T: PDL-1 treated, 2C: L-4076 control and 2T: L-4602 treated. [file 12870_2022_3489_MOESM8_ESM.docx]

| **C1-T1** | | | | |
| --- | --- | --- | --- | --- |
| **Gene Id** | **Gene_Id_Description** | **Log 2 Fold**  **Change** | ***p*-value** | ***p*adj** |
| DN15038_c0_g1_i5 | Proline dehydrogenase 2, mitochondrial | 8.93 | 9.28E-05 | 0.009105597 |
| DN24671_c0_g1_i2 | MLP-like protein 423 | 11.48 | 2.11E-11 | 2.00E-07 |
| DN32438_c1_g1_i5 | Cyclin-dependent kinase G-2 | -4.32 | 5.12E-08 | 4.04E-05 |
| **C2-T2** | | | | |
| DN29318_c0_g1_i1 | Inactive poly [ADP-ribose] polymerase RCD1 | 9.18 | 2.27E-08 | 2.36E-05 |
| DN25399_c0_g1_i3 | Probable xyloglucan endotransglucosylase | -24.08 | 2.63E-08 | 2.60E-05 |
| DN23018_c0_g1_i2 | Ferritin-2, chloroplastic | -24.28 | 2.01E-08 | 2.35E-05 |
| **T1-T2** | | | | |
| DN33722_c0_g1_i2 | Auxin transport protein BIG | 9.61 | 0.013898946 | 0.435426419 |
| DN31703_c0_g1_i2 | ABC transporter G family member 25 | 7.29 | 0.001053009 | 0.174482246 |
| DN24304_c0_g1_i1 | Protein ZINC INDUCED FACILITATOR-LIKE 1 | -6.80 | 1.62E-06 | 0.00272401 |
| DN31631_c0_g2_i1 | Probable serine/threonine-protein kinase SIS8 | -6.79 | 0.002642037 | 0.251888918 |

Supp Table 3: List of Primers from different combinations used for RT-PCR to validate NGS expression data
